# ENIGMA’s advanced guide for parcellation error identification (EAGLE-I): An implementation in the context of brain lesions

**DOI:** 10.1016/j.mex.2025.103482

**Published:** 2025-07-04

**Authors:** Evelyn Deutscher, Emily Dennis, Jake Burnett, Lyndon Firman-Sadler, Annalee L. Cobden, Michael Pink, Finian Keleher, Emma Read, Courtney McCabe, Janine Lyons, Frank G. Hillary, Elisabeth A. Wilde, Carrie Esopenko, Ekaterina Dobryakova, Andrei Irimia, Ahmed M. Radwan, Phoebe Imms, Adam Clemente, Paul Beech, Mohammadreza Mohebbi, Alex Burmester, Juan F Domínguez D, Neda Jahanshad, Sophia I. Thomopoulos, Paul M. Thompson, Karen Caeyenberghs

**Affiliations:** a Cognitive Neuroscience Unit, School of Psychology, Deakin University, Burwood, Australia; b TBI and Concussion Center, Department of Neurology, University of Utah, Salt Lake City, UT, United States; c George E. Wahlen Veterans Affairs Medical Center, Salt Lake City, UT, United States; d Department of Emergency Medicine, St. Vincent’s Hospital, Melbourne, Australia; e Turner Institute for Brain and Mental Health, School of Psychological Sciences, Monash University, Melbourne, Australia; f Department of Psychology, Penn State University, State College, PA, United States; g Department of Neurology, Hershey Medical Center, PA, United States; h H. Ben Taub Department of Physical Medicine and Rehabilitation, Baylor College of Medicine, Houston, TX, United States; i Department of Rehabilitation and Human Performance, Icahn School of Medicine at Mount Sinai, NY, United States; j Center for Traumatic Brain Injury, Kessler Foundation, East Hanover, NJ, United States; k Rutgers New Jersey Medical School, Newark, NJ, United States; l Ethel Percy Andrus Gerontology Center, Leonard Davis School of Gerontology, University of Southern California, Los Angeles, CA, United States; m Alfred E. Mann Department of Biomedical Engineering, Andrew & Erna Viterbi School of Engineering, University of Southern California, Los Angeles, CA, United States; n Department of Quantitative & Computational Biology, Dana and David Dornsife College of Arts & Sciences, University of Southern California, Los Angeles, CA, United States; o Centre for Healthy Brain Aging, Institute of Psychiatry, Psychology & Neuroscience, King's College London, UK; p Department of Imaging and Pathology, Translational MRI, KU Leuven, Leuven, Belgium; q Health and Human Sciences, Faculty of Health Sciences, School of Behavioural, Australian Catholic University, Melbourne, Victoria, Australia; r Department of Radiology and Nuclear Medicine, The Alfred Hospital, Melbourne, Victoria, Australia; s Biostatistics Unit, Faculty of Health, Deakin University, Geelong, Australia; t Imaging Genetics Center, Mark and Mary Stevens Neuroimaging and Informatics Institute, Keck School of Medicine, University of Southern California, Los Angeles, CA, USA

**Keywords:** Magnetic resonance imaging, Parcellation, Traumatic brain injury, FreeSurfer, Quality checking, visual checking, Lesion filling, Image inpainting

## Abstract

Cortical parcellation is a critical step in several neuroimaging pipelines, yet even in high quality images without pathology, errors are common. For patients with moderate to severe traumatic brain injury (ms-TBI), typical parcellation errors can be exacerbated by focal pathology impacting cortical regions. Careful visual quality checking (QC) of parcellation images of ms-TBI patients should be routinely conducted to identify the presence of parcellation errors across regions (*region error identification*). Researchers must also determine if the amount of error identified warrants the exclusion of that image (*image quality rating*). However, previous QC methods have applied somewhat ambiguous rules for region error identification and inconsistent thresholds for image quality ratings.

We developed *ENIGMA’s Advanced Guide for parceLlation Error Identification* (EAGLE-I) - a detailed training resource for identifying, classifying, and recording different error types within each individual region.

Region level errors are identified by: (a) type, *unconnected* (affecting a single ROI) or *connected* (affecting multiple ROIs); (b) size (*minor, intermediate*, or *major*); and (c) directionality, *overestimation* or *underestimation*.

Region level errors are recorded on a user friendly customised spreadsheet with standardised coding that subsequently enables automatic calculation of brain quality rating (*pass, minor error, major error, fail*, or *discuss*).

## Graphical abstract

**Figure:**
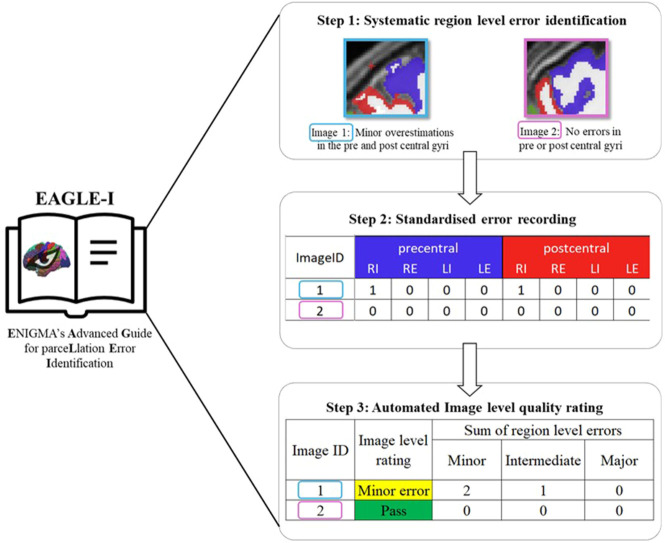

**Specifications table**

**Table:** 

**Subject area**	Neuroscience
**More specific subject area**	Brain parcellation visual quality control
**Name of your method**	ENIGMA’s Advanced Guide for Parcellation Error Identification (EAGLE-I)
**Name and reference of original method**	The Enhancing Neuro Imaging Genetics through Meta Analysis (ENIGMA) consortium Cortical QC Guide 2.0 (2017) http://enigma.ini.usc.edu/protocols/imaging-protocols/
**Resource availability**	https://github.com/ENIGMA-git#enigma-smri-imaging

## Background

Anatomical brain parcellation is the process by which brain images are partitioned into discrete anatomical regions of interest (ROIs) based on prior knowledge (e.g., expertly annotated atlases) of neuroanatomical boundaries [2]. Accurate cortical parcellation is increasingly essential, particularly in clinical populations, where researchers aim to detect biomarkers of disease progression through subtle changes in brain atrophy [21] and connectivity-based network analyses [12] However, freely available automated whole-brain parcellation tools such as FreeSurfer [6] and FastSurfer [10] can produce errors (i.e., under- or overestimation of neuroanatomical boundaries), even in structurally “normal” brains [9,11]. In many clinical populations (e.g., multiple sclerosis, stroke, traumatic brain injury (TBI)), pathology introduces additional parcellation errors that can impact downstream analyses [1,14]. Reliable identification of parcellation errors via visual quality checking (QC) is therefore a critical step in neuroimaging pipelines using automated parcellation.

Many published studies using automated parcellation tools do not specify whether QC was performed [17,22]. When QC is reported, methods are often described as in-house but with limited details provided [13]. To improve transparency, some research groups have developed structured QC protocols (see Table 1), yet inconsistencies remain in their implementation, definitions, and thresholds. ENIGMA’s cortical QC guide (ENQC; [http://enigma.ini.usc.edu/protocols/imaging-protocols/]) provides extensive visual examples but does not clearly define region-level error classifications (e.g., minor, intermediate, major; see Table 2). Radwan et. al., [19] introduced a QC method alongside their lesion-filling tool, Virtual Brain Grafting (VBG) [19]. Their method, referred to here as VBG-QC clearly defines region-level errors and overall image quality ratings, however, was conducted by expert neuroradiologists and lacks sufficient visual examples for replication. To reduce the manual workload of QC, several automated tools have been developed, but their criteria and applicability vary.

**Table 1:** Comparison of available QC methods.

**QC resources**	**Link to manual**	**Atlas used**	**Region level error types**	**Brain level quality ratings**	**Clear visual examples**	**Clear ROI error distinctions**	**Clear image QC categories**
**FreeSurfer Troubleshooting guide**	https://surfer.nmr.mgh.harvard.edu/fswiki/FsTutorial/TroubleshootingData	DK	No	No	**Yes**	No	No
**ENQC Guide** 2.0 Apr 2017	http://enigma.ini.usc.edu/protocols/imaging-protocols/	DK	Minor, Major	Pass, Moderate, Fail	**Yes**	No (BanksSTS only)	No
Qoala-T * [15]	https://www.sciencedirect.com/science/article/pii/S1053811919300138	Pial and GM/WM border surfaces	Not Provided	Excellent, Good, Doubtful, Failed	**Yes**	No	No
**VBG-QC** [19]	https://www.sciencedirect.com/science/article/pii/S1053811921000082	DK	Minor, Intermediate, Major	Good, Acceptable, Fair, Poor	No	**Yes**	**Yes**
AutoQC * [7]	https://github.com/USC-IGC/FreeSurfer_Cortex_AutoQC	DK	Pass, Fail	NA	NA	Yes	No
VisualQC * [20]	https://github.com/raamana/visualqc/blob/master/docs/VisualQC_TrainingManual_v1p4.pdf	DK	Minor, Major	Good, Minor error, Major Error, Fail, I’m tired, review later	**Yes**	**Yes**	**Yes**
**EAGLE-I** (v1.1)	LINK	DK or DKT	Minor, Intermediate, Major	Pass, Minor Error, Major Error, Fail, Discuss	**Yes**	**Yes**	**Yes**

NOTE: Quality Checking (QC) resources are listed in chronological order. * indicates an automated or semi-automated QC tool. ENQC = ENIGMA’s Cortical QC Guide, DK = Desikan Killiany, GM = Grey Matter, WM = White Matter, VBG-QC = Virtual Brain Grafting Quality Checking, BanksSTS = Banks of Superior Temporal Sulcus, DKT = Desikan Killiany Tourville.

**Table 2:** Summary of the main steps within the EAGLE-I method.

Qoala-T [15] predicts overall image quality but does not assess region-level errors, while VisualQC allows both region- and image-level assessments but relies on static snapshots, which may miss errors near focal lesions [20]. Manual QC ratings using ENQC were recently used to train AutoQC, a tool that provides pass/fail classifications for each region but does not assign an overall image rating [7]. It is possible that AutoQC could inherit inconsistencies from ENQC classifications which do not use strict thresholds for region-level error identification. Despite advances in automated QC, human reviewers remain essential for verifying error predictions, particularly in images with focal lesions, which have not yet been included during training of automated QC tools. Standardised training is especially important for researchers with minimal experience and for multicenter studies to ensure consistent error identification across raters and sites. In this paper, we introduce ENIGMA’s advanced guide for parcellation error identification (EAGLE-I), a new QC procedure designed to integrate the strengths of previous methods while improving clarity in region-level error classification and image-quality ratings.

## Method details

EAGLE-I v1.1 was developed as part of a previous lesion simulation study investigating FastSurfer parcellation accuracy in patients with moderate to severe TBI (ms-TBI; [4]. EAGLE-I has been developed to build upon previous QC procedures integrating their strengths while addressing collective weaknesses (see Table 1).

At its core, EAGLE-I is a systematic procedure for identifying, classifying and recording parcellation errors within each individual brain region. EAGLE-I is designed for use on cortical parcellations produced using either FreeSurfer or FastSurfer (Desikan-Killiany [DK] and Desikan-Killiany-Tourville [DKT] atlases respectively). The principles of region level error identification can easily be applied to subcortical regions such as the thalamus and to other parcellation schemes (e.g., Automated Anatomical Labelling (AAL) atlas)(these adaptations will be explicitly available in future versions).

EAGLE-I is a comprehensive and visually detailed resource catering to individuals with different levels of experience in QC of brain parcellations. Detailed instructions are provided, from how to load parcellation images, to how to identify and classify complex errors affecting multiple regions. Following the EAGLE-I method ensures that all brain regions are inspected in turn, and for each ROI, a decision is made as to whether an error is present. Accompanying EAGLE-I, a customised spreadsheet– referred to as the EAGLE-I error tracker is provided, along with predefined error codes, to ensure that the error classifications for each region are recorded in a standardised manner. Table 2 provides a step-by-step outline of the EAGLE-I method.

QC is prone to both inter- and intra-rater variability [15,20]. In other words, different raters may identify different errors, and the same rater might identify different errors on the same image at different times. EAGLE-I provides three key advancements to reduce this variability:

1. EAGLE-I highlights what should *not* be classified as an error, by providing clear visual examples of normal neuroanatomical variation, illusions of error, and acceptable errors.
2. Region level error sizes are based on an estimated percentage of the region affected. This provides raters with a criterion to inform their decisions.
3. The EAGLE-I error tracker automatically sums the region level error counts to determine the image level quality rating, reducing intra-rater variability in decisions.

EAGLE- I allows future users to adapt thresholds on the error tracker to suit their specific data and analyses. When using EAGLE-I within a study, an accompanying statement such as the following should be included when writing the methods sections of resultant publications:

- *‘Visual Quality Checking of Parcellation images was performed by X.X. (initials of person/s conducting the QC) according to EAGLE-I version 1.1 (reference this publication)’, AND*
- *‘Default settings for image level quality ratings were used, resulting in X images deemed to be of insufficient quality to include’, OR*
- *Thresholds for brain level image quality ratings were adapted, with parcellation images in this study deemed to be of insufficient quality to include if they met the following criteria: >’X’ minor errors, and/or ‘X’ intermediate and/or ‘X’ major errors’.*

We present EAGLE-I as a critical step towards greater transparency in neuroimaging studies using brain parcellation. Version 1.2 of EAGLE-I is already under development. At this stage, the proposed updates will not affect the fundamental processes of error identification on which the validation for EAGLE-I v1.1, presented in this paper, was conducted.

## Method validation

### Image acquisition and participant information

The images utilised in this study were part of a larger-scale dataset shared within the ENIGMA adult and paediatric ms-TBI working groups [3,18]. Informed consent was provided by participants in accordance with local ethics guidelines and the privacy rights of subjects have been preserved during data analysis under the approval of Deakin University Ethics number 2023-267. T1-weighted (T1w) scans were acquired for both healthy controls (*N* = 140) and individuals in the chronic phase of ms-TBI (*N* = 14). The MRI scans were used as input for a previous lesion simulation study (for details, see [4]) whereby 10 healthy control images were transformed to align with each ms-TBI image. This process created the following three groups of images: 1. Healthy, *lesion-free* images, 2. Simulated *lesioned* images (where the lesion was extracted from the original ms-TBI image and inserted onto the *lesion-free* image), and 3. *lesion-filled* images (where lesioned regions in the *lesioned* images) were replaced with signal intensities estimating healthy tissue using Virtual Brain Grafting (VBG; [19]). One lesion profile did not simulate successfully and as such the 10 images corresponding to that image were excluded from this study. A subset of 39 images (10%) of the total 390 images generated in the initial study were used for validation of EAGLE-I. The 39 images were chosen by pseudo-random selection, such that equal numbers of each image type (*lesion-free, lesioned* and *lesion-filled*) were included and each of the 13 remaining ms-TBI lesion profiles were represented within each image type. All image processing was performed using the Multi-modal Australian ScienceS Imaging and Visualization Environment (MASSIVE), M3 High Performance Computing (HPC) facility [8].

### Image parcellation

Cortical parcellation for all T1-w scans was performed using FastSurfer’s (v1.0.0) *recon-surf* pipeline [10]. Each *recon-surf* run was allocated a runtime of 2.5 hours. All images successfully completed parcellation on the first run.

### Blinding procedure

To remove parcellation errors occurring within lesioned areas, lesion masks were used to remove any parcellations that fell within the lesioned area. Lesion masks were similarly imposed onto *lesion-free* images. This was done to ensure raters using EAGLE-I were blind to the image type during error identification.

### EAGLE-I training and implementation

A total of nine raters across two sites, Deakin University, Australia (ED, JB, LF-S, AC) and University of Utah, United States (MP, FK, EN, CM and JL) were trained using the EAGLE-I method developed for this study (https://github.com/ENIGMA-git#enigma-smri-imaging). The nine raters had a background in psychology or neuroscience but limited, if any, prior experience in conducting QC of parcellation images. Training consisted of two virtual seminars led by ED. All raters then participated in three practice rounds consisting of QC of two parcellation images. After completing the practice rounds, another virtual meeting was held during which overall and personalised feedback was provided. As a result of discussion and feedback from these sessions, several suggestions (i.e., informing decisions according to number of slices affected) were immediately incorporated into the original EAGLE-I document (v1.0). Therefore, EAGLE-I v1.1, presented in this paper, reflects the full extent of training provided to the raters. After completion of the final feedback round and incorporation of resulting updates in the protocol, the nine raters then conducted QC for the same set of 39 parcellation images, referring to EAGLE-I v1.1 as much as needed to aid in decision making.

### Statistical analysis

Three main statistical analyses were performed to assess the validity of EAGLE-I. Firstly, we explored the frequency and type of regional errors in FastSurfer cortical parcellations, using an ordered logistic regression to examine whether image type (*lesion-free, lesion-filled*, or *lesioned*) influenced image-level quality ratings. Secondly, we assessed the consistency of region-level error identification among raters trained using EAGLE-I by calculating Cronbach’s alpha correlations for each region. Lastly, we calculated intraclass correlation coefficients (ICCs) to identify the agreement among raters for image level quality ratings. All statistical analyses were performed in R version 4.2.2 (2022-10-31), within RStudio 2022.12.0+353, with results considered significant at p ≤ .05.

### Exploration of error types and frequency in FastSurfer cortical parcellation

**Fig. 1:**
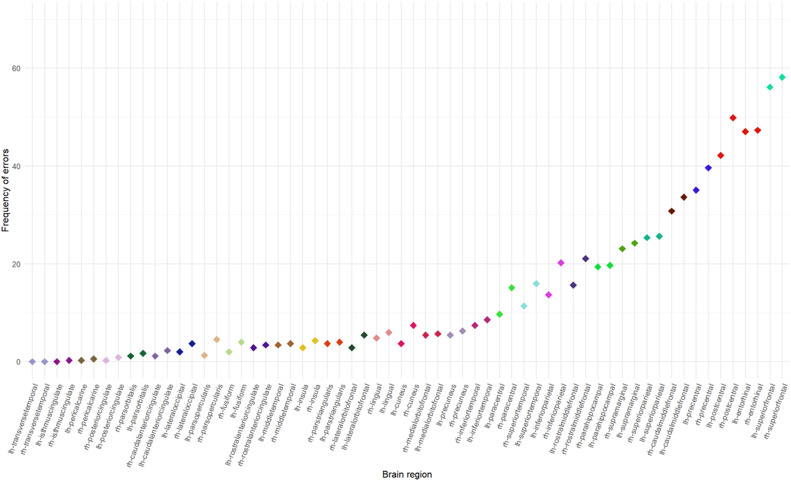
Frequency of all errors identified in each brain region.

**Fig. 2:**
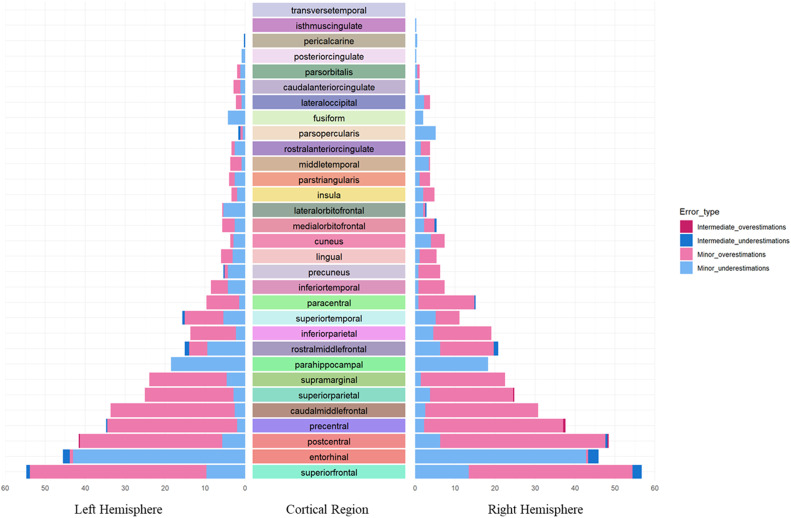
Frequency of error types and sizes identified within brain regions.

The combination of nine raters performing QC on 39 images produced 351 QC ratings for analysis. Each individual region (*N* = 21,762 [resulting from 62 regions in each of the 351 ratings]) was identified as having either no error (*n* = 18,913), minor error (*n* = 2,797), intermediate error (*n* = 52) or a major error (*n* = 0). The frequency of any errors observed for each cortical region are shown in Fig. 1, and the frequency of error types within regions is shown in Fig. 2. Only three regions, the *left transverse temporal, right transverse temporal* and *right isthmus cingulate* were error-free across all raters and all images. Bilaterally, the superior frontal *cortex* was found to have the highest frequency of errors, with errors identified over 50% of the time (right 58%, left 56%). Errors were identified bilaterally in five other regions over 25% of the time (including the *entorhinal cortex, postcentral gyri, precentral gyri, caudal middle frontal gyr*i*, and superior parietal cortex*).

An ordered logistic regression analysis was run to determine whether image level quality ratings differed based on image type (*lesion-free, lesion-filled or lesioned*). Image type was treated as an ordinal predictor (coded as 1, 2, and 3) based on the increasing presence of lesion content. Similarly, the image-level quality rating (pass, minor error, major error, or fail) was treated as an ordinal response variable, reflecting a progression in error severity. The model did not show a significant relationship between image type and image level quality ratings (*p* = 0.47). Comparing odds ratios for *lesion filled* (0.89) 95% CI [.71, 1.12] and *lesioned* (0.88) 95% CI [.65, 1.18] images also showed their error classifications were not significantly different from that of the ground truth *lesion free* images (*p* = .361 and *p* = 0.396 respectively). The overall model was not statistically significant based on the Wald chi-square test χ²(2, N = 351) = 1.48, *p* = .477. As a result, subsequent analyses were run with all image types combined.

### Consistency of regional level error identification among raters

**Fig. 3:**
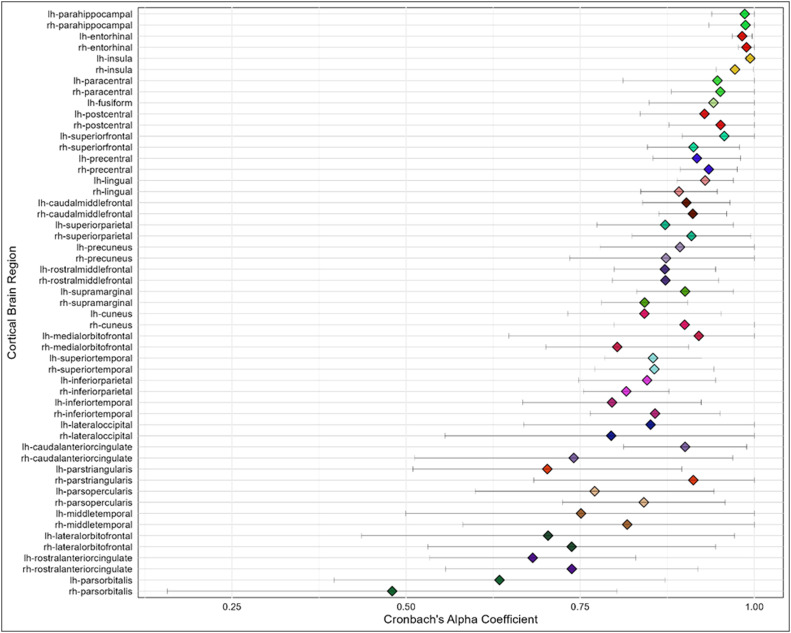
Cronbach alpha coefficients calculated across the nine raters for each brain region with sufficient variance.

Of the 62 cortical regions within each of the 39 images (*n* = 2,418) the nine raters were in total agreement of ‘no error’ in more than half (54%) of the regions (*n* = 1,296). For the remaining regions in which at least one rater identified an error (*n* = 1,122), Cronbach alpha correlations were calculated to identify consistency among raters’ identification of any error or no error (insufficient variance existed for investigation of major or intermediate errors). Fig. 3 shows the range of Cronbach alpha correlations for each region; upper CI intervals were truncated to 1 (where rounding errors pushed them over; minimum = 0.48, maximum = 1). Following common interpretations of Cronbach’s alpha [5], values above 0.9 indicate excellent consistency, 0.8–0.89 indicate good consistency, and 0.7–0.79 acceptable, 0.6-0.69 questionable and <0.6 poor consistency. The average correlation taken across all regions was 0.86 indicating a good consistency of regional error identification between raters.

### Absolute agreement among raters for image level quality ratings

Of the 39 images included in this study, no images obtained absolute agreement between the raters on image level quality rating. Inter-rater agreement for image level quality rating was addressed by calculating intraclass correlation coefficients (ICC) based on a single rating, absolute-agreement two-way random-effects model [16]. The 5-point image quality rating, ICC(A,1) = 0.22, *p* < 0.001, (0.12, 0.35) revealed a poor to fair agreement between raters. In line with previous research [15], we investigated whether reducing the subject level ratings from a 5-point (Pass, Minor Error, Major Error, Fail, Discuss) to either a binary (Include, Exclude) or a 3-point classification (Pass, Error, Fail) would improve agreement. The binary rating ICC(A,1) = 0.20, *p* < 0.001, (0.11, 0.33) resulted in poor agreement while the 3-point ICC(A,1) = 0.24, *p* < 0.001, (0.14, 0.38) confidence intervals ranged from poor to fair. Comparison of *Z* scores revealed no significant differences between the 5-point and 3-point ICC scores (*Z* = 0.33, *p* = 0.53), the 5-point and binary ICC scores (*Z* = 0.33, *p* = 0.74), or between the 3-point and binary ICC scores (*Z* = 0.63, *p* = 0.53).

### Limitations

EAGLE-I’s main limitation is the lack of agreement for image-level quality ratings, which likely stems from strict thresholds where one minor error can shift an image's quality rating. In EAGLE-I v1.1, we have addressed this by informing users of their option to change thresholds to suit the analysis they will run. If changes to the threshold settings are adequately reported within future publications, the use of EAGLE-I will still provide confidence that errors at the individual region level are being consistently identified. Secondly, it is possible that misclassification errors (i.e., where the grey matter boundary has been respected, but the regional neuroanatomical boundary may have shifted), may be under-represented in our error counts. Detection of these errors requires an in-depth understanding of neuroanatomy. While EAGLE-I v1.1 included detailed examples for the common regions where misclassification errors occur, the next version will provide examples across a wider range of regions to improve the identification and recording of these types of errors.

In conclusion, FastSurfer’s advanced whole brain parcellation pipeline shows vulnerability to producing parcellation errors in both healthy images and images with pathology. The persistence of these errors requires a coordinated and consistent approach to their identification and recording, to ensure they are appropriately accounted for in subsequent analyses. Our proposed method, EAGLE-I, incorporates both a comprehensive training resource for region error identification, along with automated image level quality ratings. EAGLE-I is a valuable resource for facilitating consistent region error identification across multiple raters with little to no prior experience in visual quality checking of cortical parcellation images.

## Ethics statements

Informed consent was provided by participants in accordance with local ethics guidelines and the privacy rights of subjects have been observed with data analysis occurring under the approval of Deakin University Ethics number 2023-267.

## Declaration of generative AI and AI-assisted technologies in the writing process

During the preparation of this work the primary author (ED) used ChatGPT to edit small sections of the text to improve its clarity and conciseness. After using ChatGPT, the author reviewed and further edited the content and takes full responsibility for the content of the publication.

## Declaration of competing interest

The authors declare that they have no known competing financial interests or personal relationships that could have appeared to influence the work reported in this paper.

## Data Availability

De-identified data from this study are not publicly available, the data could be made available by joining the ENIGMA brain injury working group and agreeing to its Memorandum of Understanding

## Appendix Supplementary materials

Image, application 1
