# Supplementary material for: ENIGMA’s advanced guide for parcellation error identification (EAGLE-I): An implementation in the context of brain lesions
Source: MethodsX. 2025 Jul 4;15:103482. doi: 10.1016/j.mex.2025.103482 (PMC12335952; doi:10.1016/j.mex.2025.103482)
Supplement: Supplementary file 1 [file mmc1.pdf]

ENIGMA's **A**dvanced **G**uide for parce**L**lation **E**rror **I**dentification

EAGLE-I

(v1.1 March 2025)

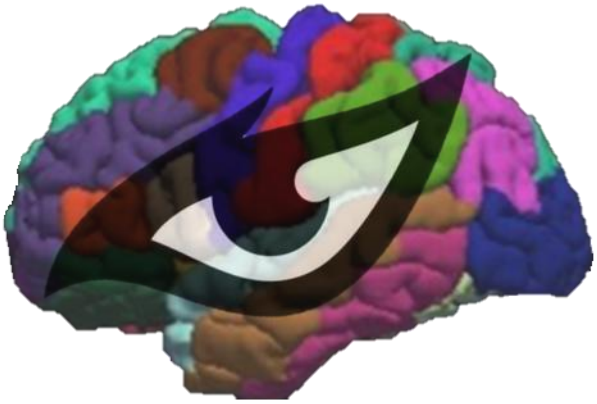

Contents:

Acknowledgments and version control 3
1.0 Introduction
1.1 Pre-amble 4
1.2 Summary of previous cortical parcellation QC protocols (recommended pre-reading) 4
2.0 What constitutes an error? 5
2.1 True errors vs. normal neuroanatomical variation 5
2.2 Illusions of Error 7
2.3 Acceptable Errors 9
2.4 Informing decisions according to number of slices affected 10
2.5 Summary of true errors, illusions of error and acceptable errors 11
3.0 Classifying Errors 11
3.1 *Connected* and *Unconnected* Errors 12
3.2 FreeSurfer Common Error Regions and Caution Regions 13
3.4 Mislabeling Errors 14
3.3 Classifying Error types 15
3.3.1 Minor Errors 15
3.3.2 Intermediate Errors 17
3.3.3 Major Errors 18
3.3.4 Control Point Errors 19
4.0 Recording Errors 19
4.1 EAGLE-I ET 19
4.2 Code for Recording Errors in Spreadsheet 20
5.0 Image QC Ratings 22
5.1 Discussion of CP Fix Errors 23
6.0 Loading FastSurfer Volumes, Surfaces, and Lesion masks in Freeview 23
6.1 Loading from Bash Command 23
6.2 Manual loading 24
6.3 Turn off WM parcellations (Optional) 25
6.4 26
7.0 Spreadsheet Formulas for automatically adding ROI level errors 26

Acknowledgments and version control

We would like to thank the following people who spent countless hours using EAGLE-I to perform QC. Their experiences and feedback have been invaluable for shaping this resource

- Jake Burnett
- Lyndon Firman-Sadler
- Annalee Cobden
- Michael Pink
- Finian Keleher
- Emily Nilsson
- Courtney McCabe
- Janine Lyons

Version number	Release Date	Description of updates	Person responsible	Review date
1.1	03/2025	Polished formatting ready for public release	EMD	09/2025
1.0	06/2022	Initial Version	EMD	

1.0 Introduction

1.1 Preamble

EAGLE-I aims to remove some of the ambiguity and inter-rater bias that dominates visual quality checking (QC) of cortical parcellations in neuroimaging. Here we present:

1) a systematic method for the search and identification of errors,
2) clear rules for classifying and recording errors in each brain region, and
3) automated brain level quality ratings using region level error counts

This guide should be read in full when first training to conduct QC, but thereafter, links in the contents can provide quick reference to specific sections as required. For ease of formatting, many images have been shrunk, we highly recommend zooming in to appreciate finer details. Images with a variety of quality and contrast have been included to provide a better representation of the variation encountered when performing QC. For those who have never conducted QC before, advancing to section 6 of this resource will provide detailed descriptions of how to load parcellations so that you can follow along with the examples provided.

1.2 Summary of previous cortical parcellation QC protocols (recommended pre-reading)

EAGLE-I is created in addition to four QC resources previously developed (see Table 1) and is not designed to be used as a stand-alone QC guide. Before implementing this protocol, we recommend users read the ENIGMA Cortical QC (ENQC) guide and the FreeSurfer tutorial. EAGLE-I combines the strengths of previous methods whilst addressing collective limitations.

Table 1. Summary of previous Cortical QC parcellation protocols

	Link to manual	Atlas used	Region level error types	Brain level quality ratings	Clear visual examples	Clear ROI error distinctions	Clear image QC categories
**FreeSurfer Troubleshooting** guide	https://surfer.nmr.mgh.harvard.edu/fswiki/FsTutorial/TroubleshootingData	DK	No	No	**Yes**	No	No
**ENQC** Guide 2.0 Apr 2017	http://enigma.ini.usc.edu/protocols/imaging-protocols/	DK	Minor, Major	Pass, Moderate, Fail	**Yes**	No (BanksSTS only)	No
**Qoala-T** (Klapwijk et al 2019)	https://www.sciencedirect.com/science/article/pii/S1053811919300138	Pial and GM/WM border surfaces only	Not Provided	Excellent, Good, Doubtful, Failed	**Yes**	No	No
**VisualQC** (Reddy Raamana, 2023)	https://github.com/raamana/visualqc/blob/master/docs/VisualQC_TrainingManual_v1p4.pdf	DK	Minor, Major	Good, Minor error, Major Error, Fail, I'm tired, review later	**Yes**	**Yes**	**Yes**
**VBG-QC** (Radwan et al., 2021)	https://www.sciencedirect.com/science/article/pii/S1053811921000082	DK	Minor, Intermediate, Major	Good, Acceptable, Fair, Poor	No	**Yes**	**Yes**
**EN_AutoQC** (Gadewar et al., 2021)	https://github.com/USC-IGC/FreeSurfer_Cortex_AutoQC	DK	Pass, Fail	NA	NA	Yes	No
**EAGLE-I** (v1.1)		DK or DKT	Minor, Intermediate, Major	Pass, Minor Error, Major Error, Fail, Discuss	**Yes**	**Yes**	**Yes**

NOTE: ENQC = ENIGMA's Cortical QC Guide, DK = Desikan Killiany, GM = Grey Matter, WM = White Matter, VBG-QC = Virtual brain grafting quality checking, BanksSTS = Banks of Superior Temporal Sulcus, DKT = Desikan Killiany Tourville.

2.0 What constitutes an error?

2.1 True errors vs. normal neuroanatomical variation

This protocol is not designed to conduct a comprehensive review of atlas alignment at all neuroanatomical borders. Our aim instead is to conduct a detailed visual quality assessment of the **accuracy of cortical parcellation as can be evidenced by visual comparison to the underlying T1w image**. We identify regions of grey matter (GM) or white matter (WM) signal intensity that have not been parcellated (underestimations), and we identify parcellations that protrude outside the GM boundary (overestimations). Referring to the underlying T1-w image prior to final error classification will allow you to understand whether an abnormal appearing region is a result of an accurate parcellation of neuroanatomical variation in the underlying T1-w image (not an error), or if the parcellation tool strayed from the underlying T1-w image resulting in the abnormal appearing parcellation (an error). There are some regions where FreeSurfer is known to struggle to produce accurate parcellations and some of these cannot easily be classified without expert neuroanatomical training, these will be outlined in section 3.2.

Normal Neuroanatomical variation

The panel of three images below, taken from both the external and internal views of the same parcellation image, highlights the need for looking across all three imaging planes, in both the internal and external views, before making a decision about whether an error is present or not.

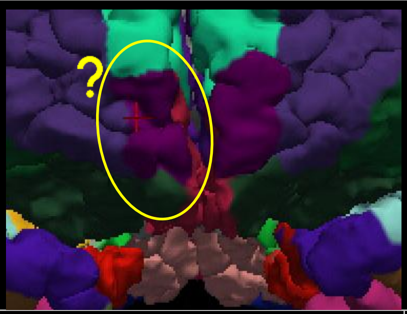

Right frontal pole appears smaller than the left frontal pole.

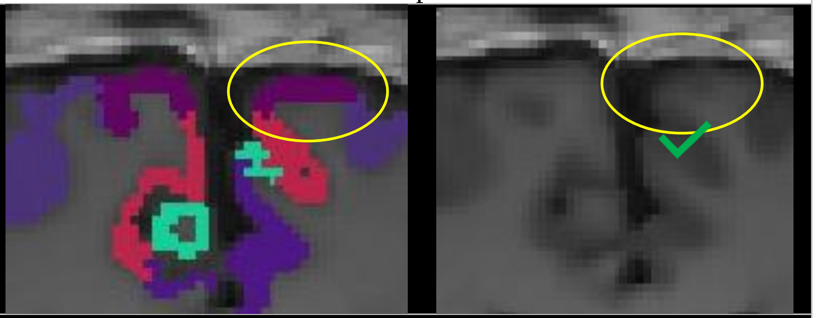

In the internal views, the parcellation is seen to accurately follow the GM boundary and therefore is not an error.

Respecting variation in Neuroanatomical boundaries

The images below show examples where the circled regions have been overestimated into other regions. Given the huge variance in normal neuroanatomy and the expertise required to identify these more subtle boundary shifts, in this version of EAGLE-I we only identify these mislabeling errors in pericalcarine and banks of superior temporal sulcus (examples provided in section 3.4).

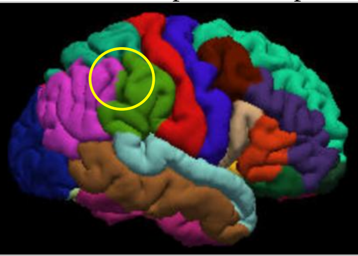

Possible overestimation of the supramarginal gyrus (green) onto the inferior parietal (pink)

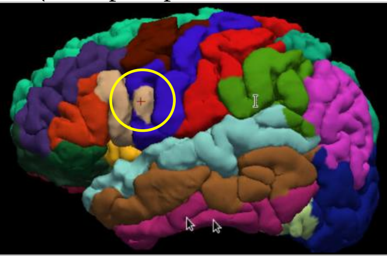

Possible overestimation of the pars-opercularis (cream) onto the precentral gyrus (deep purple)

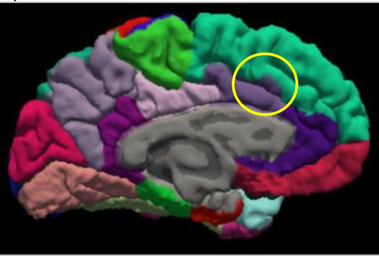

Possible overestimation of the caudal anterior cingulate (purple) onto the superior frontal (green)

Dura & Sinuses

The Images below identify several neuroanatomical landmarks that can disrupt cortical parcellation. For those new to conducting parcellation QC, these regions can often look like sections of missing (underestimated) GM. However, the true error occurs only when these landmarks are incorrectly included in parcellations, resulting in overestimations of the affected regions.

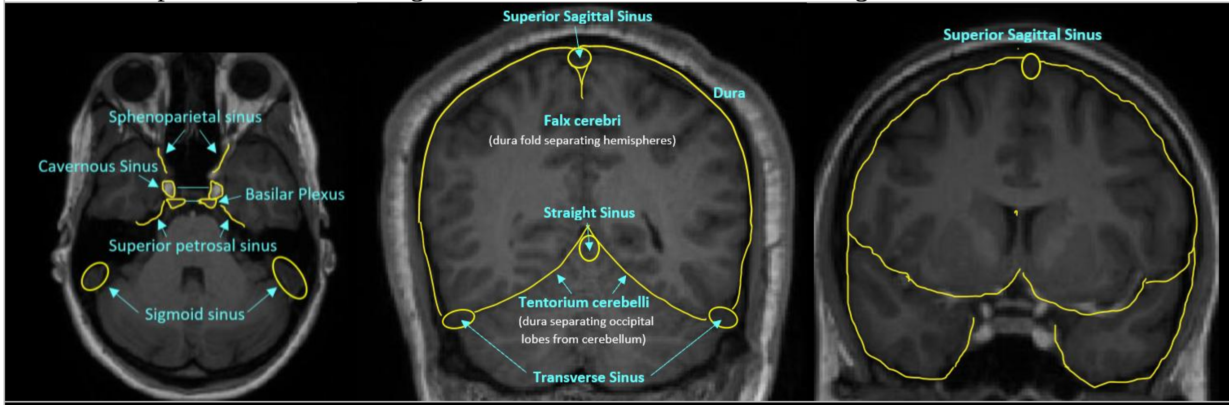

** The annotations above are not intended to be anatomically correct and should be used only as an aid to identify these regions of caution when conducting FS QC using this guide **

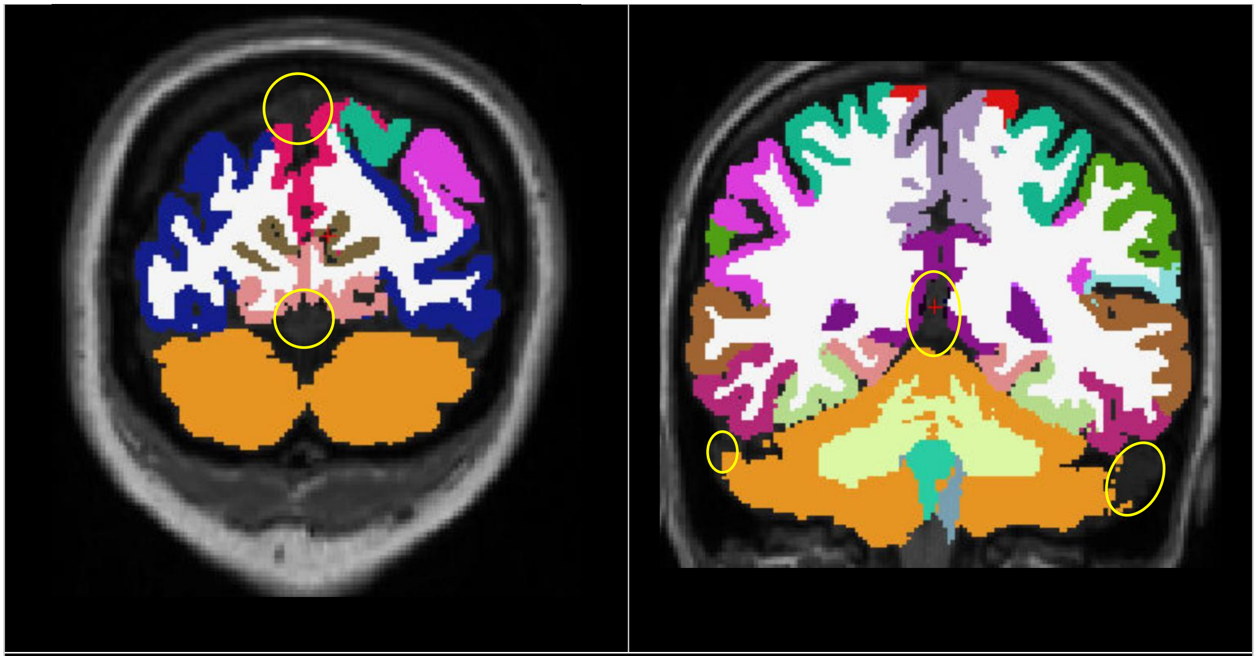

In coronal slices of the posterior portions of the brain, it is common for the lingual, occipital and cuneus to appear underestimated. The parcellations above are in fact accurate, and the regions circled correspond to sinuses.

2.2 Illusions of Error

Given that MRIs represent a 3D brain as a series of 2D slices, it is not possible to perfectly match the curvature of the brain at every slice. The examples below highlight areas where, despite the appearance of unlabeled GM regions, they are not true errors but rather classified as 'illusions of error'. These illusions generally appear only in one or two slices and are followed, and/or preceded by, accurate parcellations.

Illusions of Error

The caudal middle frontal gyrus (dark brown) is shown below in 3 sequential slices in the axial plane. The appearance of a potential error in the middle slice is due to the angle of the sulci folding away from the angle of the MRI. Moving one slice forward or back (as shown), reveals normal parcellations for this region. Therefore, the middle slice is considered an illusion of error.

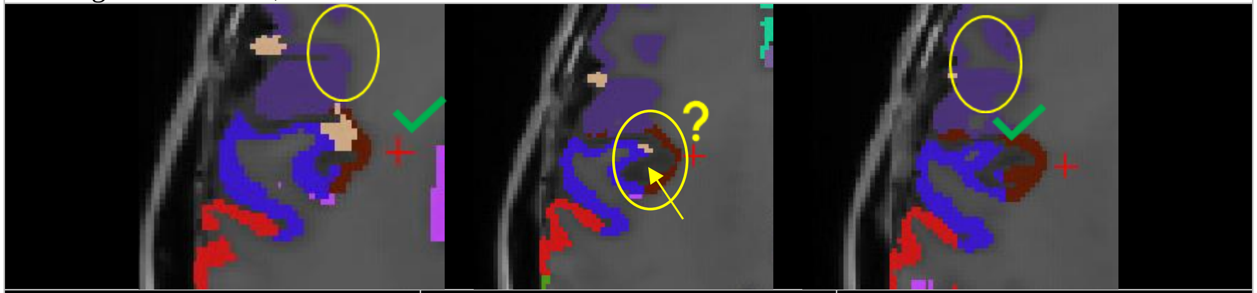

In this first slice, the region appears slightly thin but still normal.	On this 'problem' slice, the caudal middle frontal appears too thin, and an unlabeled GM can be seen	Just one slice on, the region looks thicker and normal.

The examples below highlight regions near the frontal pole, in both the coronal (left) and axial (right) planes respectively, where illusions of error are common. The regions appearing to be mislabeled are present in one slice in the left-hand images, but in the following slice (right hand images) the parcellation has 'caught up', revealing an accurate parcellation. Therefore, the apparent unlabeled regions in the first slices are considered as illusions of error rather than true error.

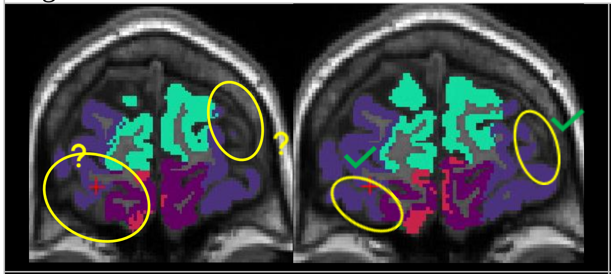

Left, questionable bilateral rostral middle frontal underestimations. Right (one slice on), the parcellations appear normal

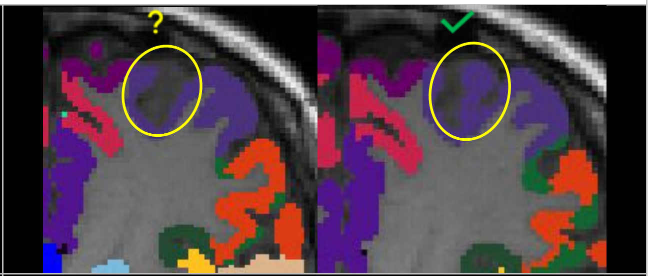

Left, possible left hemisphere rostral middle frontal underestimation. Right, one slice one, normal segmentation.

As you move posteriorly through the brain in the coronal view, several regions may commonly appear underestimated due to the angle of the sulcal folding as the MRI is slicing perpendicularly across them. The parcellation will usually 'catch up' within a slice or two.

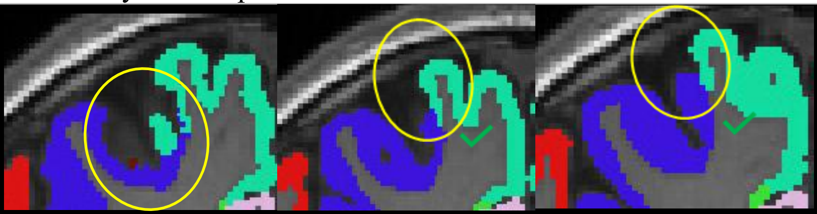

The pre-central and superior frontal regions appear possibly underestimated in the left image, but in subsequent slices appear normal. Thus, the image on the left is considered to be an illusion of error.

Sagittal slices surrounding the midline

These often show very abnormal looking parcellations due to the MRI capturing the presence of the falx cerebri (dura folding between the hemispheres) which often appears as a shadow moving across the whole brain over a period of 3-4 slices. These slices immediately to the left and right of the midline affected by this shadow effect should not be directly looked at for error identification. They can be used (with caution) as another view to back up an error identified in a different plane.

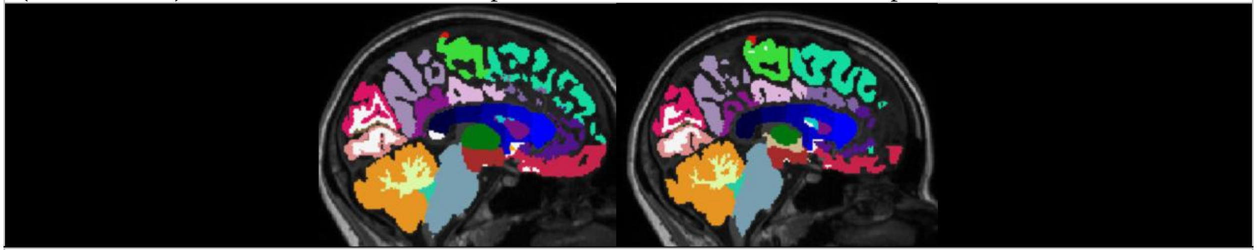

Midline sections in the coronal view

The presence of the falx cerebri and sulcal folding along the midline, in conjunction with the angle of the MRI in the coronal plane makes this region prone to illusions of error. In the anterior portion of the brain, it is common for the superior frontal, and both the rostral and caudal anterior cingulate to appear underestimated at the midline. However, this is often only an illusion of error that is resolved over a few slices as shown below.

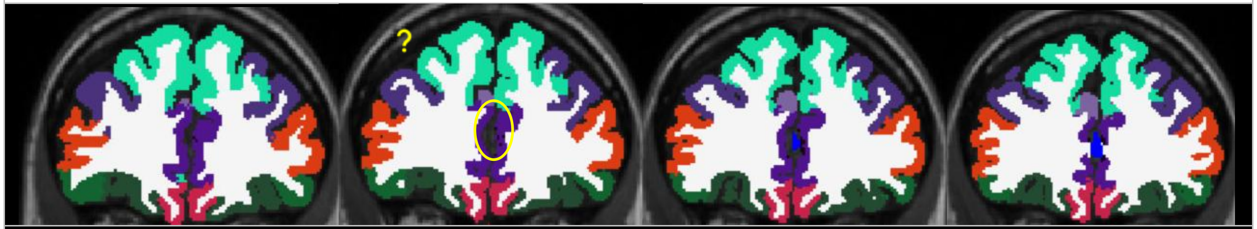

This sequential four slice sequence of images show an illusion of error in the slice second from the left, surrounded by accurate parcellations in the other slices.

Entangled Internal ROIs

It is common for parcellated regions to appear entangled as one region is disappearing while another is appearing. Although in any one slice the parcellations may look as though one is encroaching on the other’s space, scrolling slowly anteriorly to posteriorly and back again should reveal a clear transition from one region to the next (with the entanglement only occurring as a temporary overlap on the 2D image).

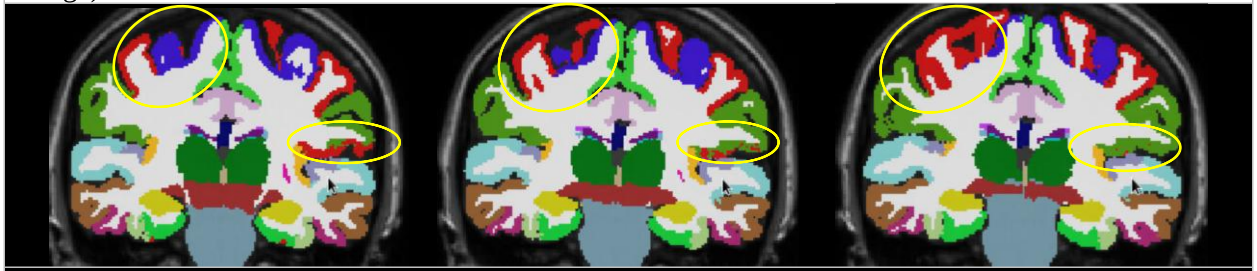

Moving left to right through these images, in the top left, the postcentral (blue/purple) is disappearing and the precentral (bright red) is appearing. In the middle right of the images the same is happening with the pars triangularis (red) and the pars orbitalis (green). These are both normal, accurate parcellations.

2.3 Acceptable Errors

In our experience, to date, there exists no such thing as a perfect parcellation. Therefore, it is important to establish what constitutes an acceptable level of error. The visual examples below represent what we deem to be acceptable errors that do not warrant recording.

**Acceptable Errors**
In the external view below, there appears to be flattening of the cortical surface of the middle temporal region. Inspection of this area on both the axial and coronal internal views reveals that the parcellation is within the dura and represents the T1 image accurately. Therefore, the cortical flattening in this instance is deemed to be an acceptable error.
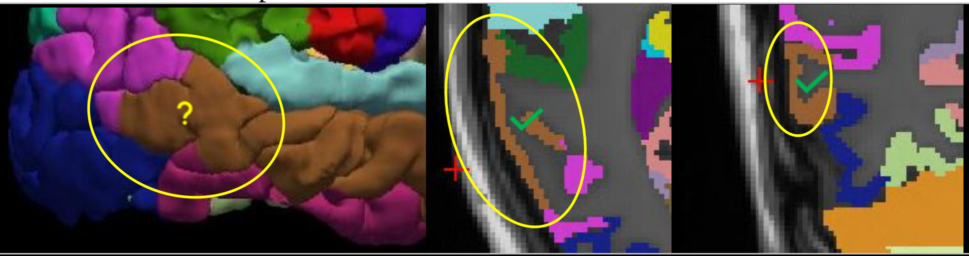
External flattening of the middle temporal (right image). Parcellation respects the GM boundary in internal views, therefore deemed acceptable (no error).

2.4 Informing decisions according to number of slices affected

There are instances where even if an error appears as a true error across all three planes, it may be so minor in relative size, or it may persist for such few slices that we deem it an acceptable error. The guidelines below are provided as an estimation only to guide you in making a decision when an error is small or persists for only a few slices. The lower limit ≥3 slices to the upper limit of 6 or more slices was adopted in line with previous literature identifying errors persisting in six or more consecutive slices (Iscan et al., 2015). You do not need to strictly adhere to these slice numbers. You should make your decision considering all 3 factors: 1) size of the error, 2) how 'true' the error is, and 3) how many slices the error persists for. The table below provides some examples and whether they were recorded as errors.

	**True error (distinct in all 3 planes)**		**Question of illusion (in one plane)**	
	**Very small**	**Minor to Intermediate**	**Very small**	**Minor to intermediate**
1 – 2 slices only	**Do Not Record**	**Do Not Record**	**Do not record**	**Do not record**
3 – 5 slices only	**Rater decision**<br>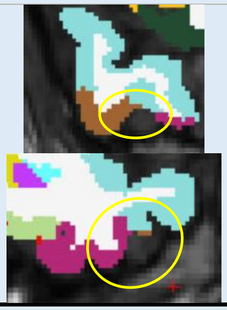	**Rater Decision**	**Do Not Record**<br>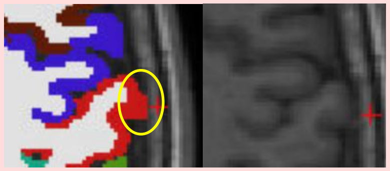<br>Possible pial overestimation, however, doesn't appear to extend all the way into the dura. External view looked ok, so this was deemed an acceptable error.	**Rater Decision**<br>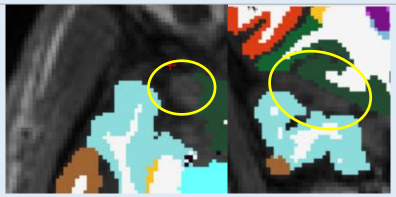<br>Axial view (left) suggests underestimation of the lateral orbitofrontal, but coronal view (right) suggests it is possibly the sylvian fissure.
6 + slices	**Rater decision**<br>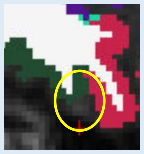	**Record**<br>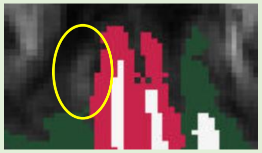		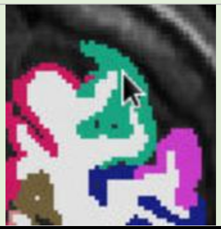

2.5 Summary of true errors, illusions of error and acceptable errors

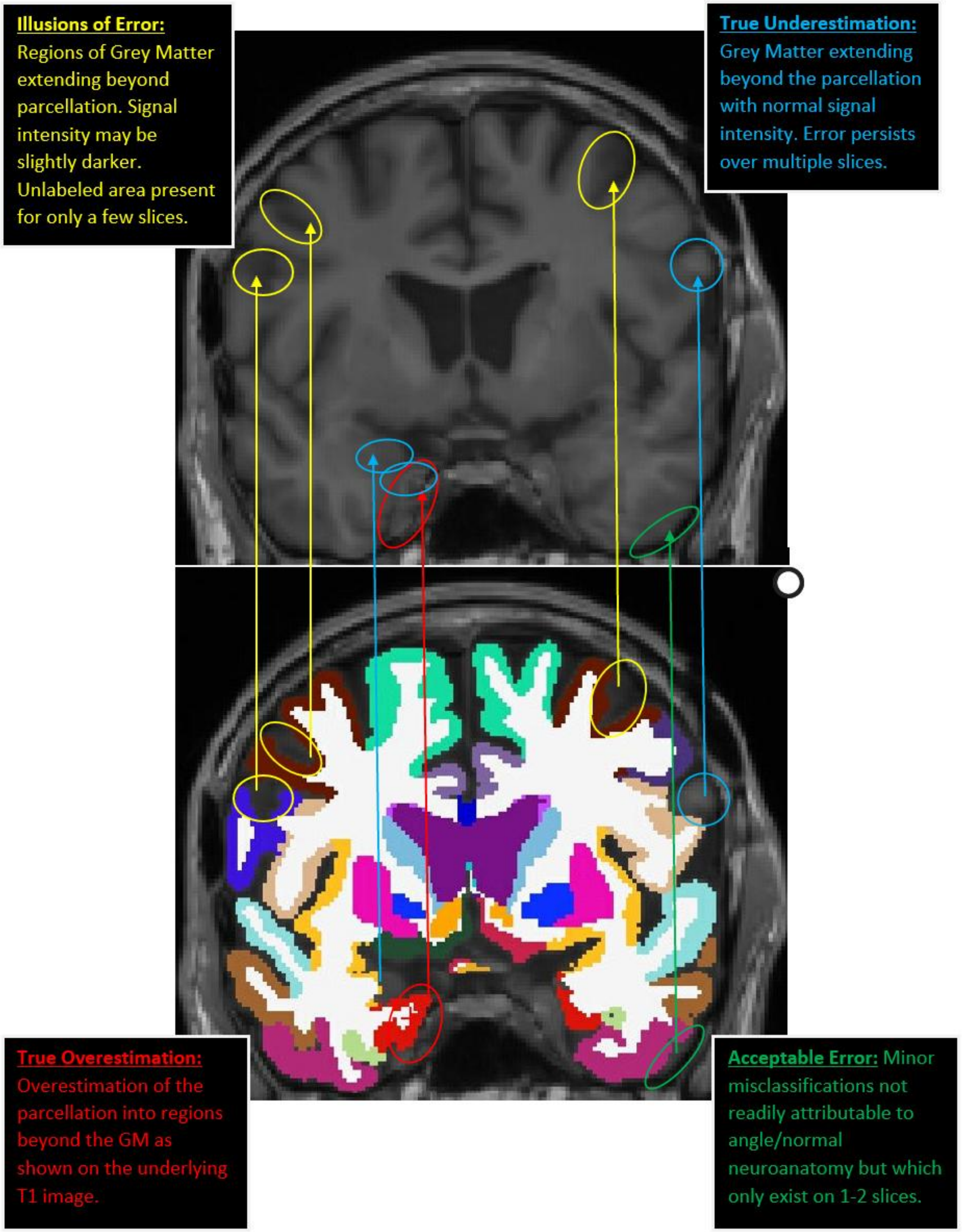

3.0 Classifying Errors

3.1 *Connected* and *Unconnected* Errors

One of the common points of confusion when implementing previous cortical QC protocols was how to deal with large errors that crossed over multiple ROI boundaries. If every affected region was counted as an error, the overall brain level error counts would be inflated, potentially leading to patient exclusion when the parcellation outside of the large error might otherwise be accurate. On the other hand, if the large, connected errors are not identified and recorded for each ROI affected, subsequent analysis could be biased in those regions. The table below details how this protocol distinguishes between connected and unconnected errors to improve consistency of classification among raters.

UNCONNECTED<br>An error restricted to one region of interest (ROI), i.e., does not cross over parcellation boundaries.		
Minor underestimation, rostral middle frontal	Minor unconnected overestimation of the inferior parietal (pink).	3 unconnected minor overestimations of the precentral (purple) & postcentral (red) and inferior parietal (pink) ROIs
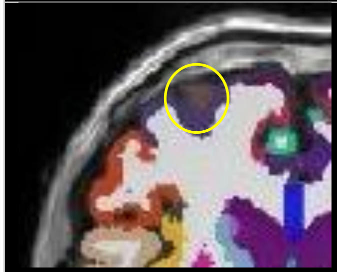	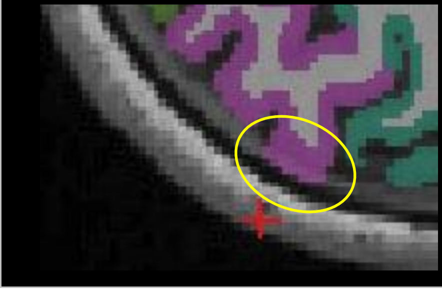	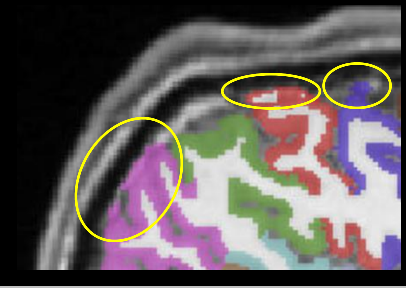
The below error consists of minor pial overestimations in both the precentral and postcentral gyri. These errors, although occurring very closely together in neighboring regions, remain as separate errors in all three planes and in the external view. They are therefore recorded as unconnected minor errors.		
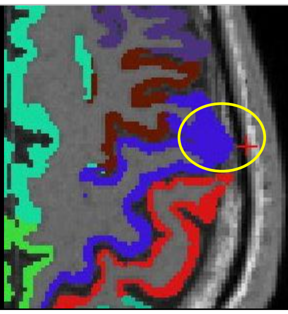 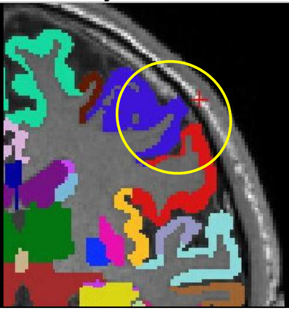 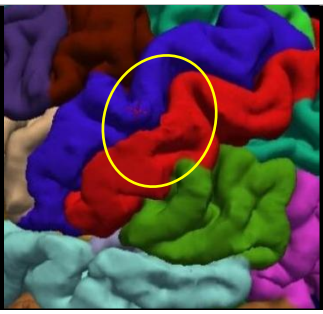		

<table>
<tr><td colspan="3">CONNECTED
When multiple errors occur at the intersection of two or more neighboring regions and they cannot be disentangled, (i.e., there is no clear point where one error starts, and the other error begins), they are classified as connected errors.</td></tr>
<tr><td>Connected underestimation at the inferior temporal (pink), superior temporal (aqua) and middle temporal (brown).</td><td>Connected minor overestimation/cortical flattening at the rostral middle frontal (purple) and pars triangularis (red)</td><td>Minor underestimation of both the frontal pole (grey) and the inferior temporal (pink).</td></tr>
<tr><td>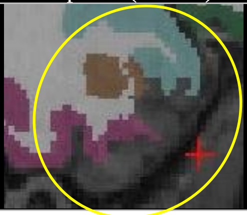</td><td>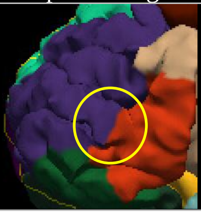</td><td>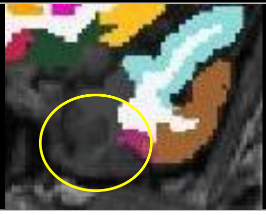</td></tr>
<tr><td colspan="3">Below: This error appears similar to the unconnected post and precentral gyri errors above. However, the error below contains an overlap between the two regions. In the external view, the error crosses over the neuroanatomical border making it difficult to distinguish where the error for one region would stop and where the error in the next would begin. For this reason, this error is classified as connected.</td></tr>
<tr><td colspan="3">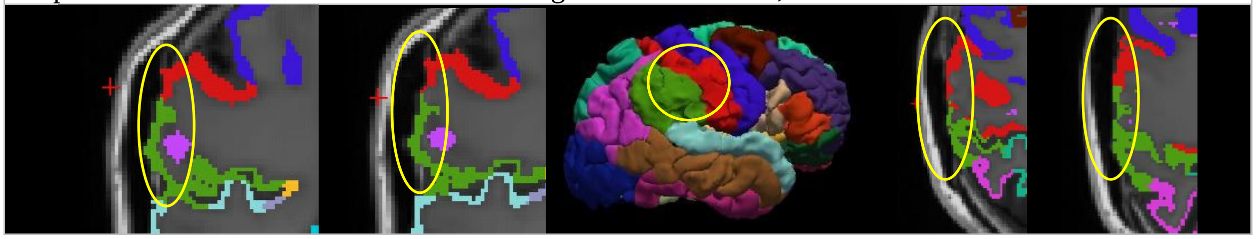</td></tr>
</table>

3.2 FreeSurfer Common Error Regions and Caution Regions

FreeSurfer is known to commonly produce errors in three regions of the DK atlas; the insula, entorhinal cortex and parahippocampal gyrus. We refer to these regions as FreeSurfer Common Error Regions (FCER). In the VisualQC paper (Reddy Raamana, 2023), it was found that only 4 from 2688 scans were free from any errors in the FCER, highlighting just how common these errors are. The ENQC guide provides detailed visual examples of these regions, additional examples are below. These regions are provided leniency when it comes to region level error classification. What might be considered intermediate errors in other regions, we allow to be classified as minor within the FCER. These regions are also treated differently when counting errors to determine brain level quality ratings (Section 5).

<table>
<tr><td colspan="3">Insula</td></tr>
<tr><td>Good parcellation (no error)</td><td>Minor underestimation</td><td>Minor underestimation</td></tr>
<tr><td>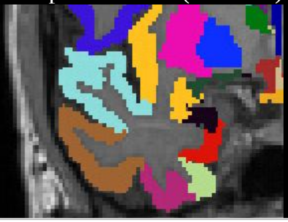</td><td>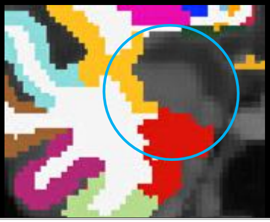</td><td>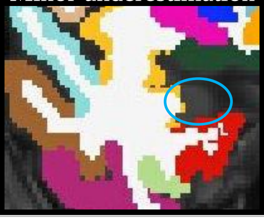</td></tr>
</table>

Entorhinal (Ent) Cortex		Parahippocampal (Para) gyrus	
Para: minor underestimation<br>Ent: minor underestimation	Para: minor underestimation<br>Ent: minor underestimation	Para: minor underestimation<br>Ent: minor underestimation	Para: minor underestimation<br>Ent: minor underestimation
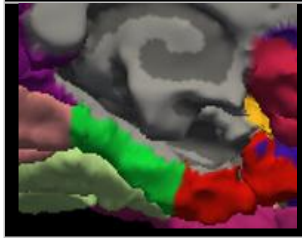	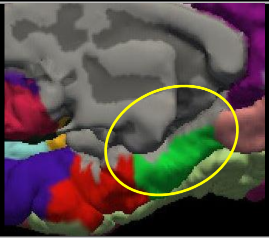	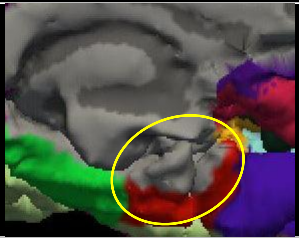	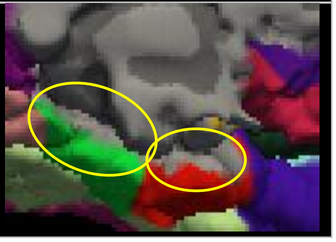

In addition to the FCER, our experience has identified other regions which frequently contain errors. Identification and classification of these errors is no different to normal, however. In addition to the temporal pole examples below, other caution regions include the pre/post central gyrus, and overestimations of the supramarginal**,** and cingulate cortex. The ENIGMA Cortical QC Guide 2.0 provides good visual examples of the type of issues common in these regions.

Temporal Pole (FreeSurfer DK ATLAS ONLY not present in FastSurfer DKT atlas)			
Good parcellation (no error)	Minor underestimation of temporal pole only	Minor underestimation of the temporal pole	Minor connected underestimation of: temporal pole (grey), inferior temporal (pink) and middle temporal (brown).
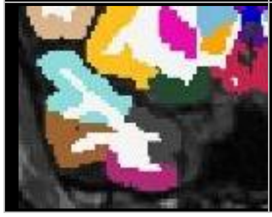	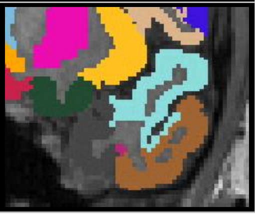	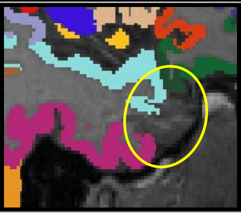	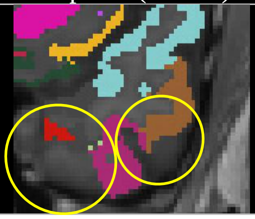

3.4 Mislabeling Errors

Although EAGLE-I focuses on identification of errors with respect to the underlying T1w image, there are two regions where mislabeling (i.e., shifts of the neuroanatomical borders of regions, while still representing accurate GM/WM segmentation) are common and easily identifiable by non-experts. Examples of mislabeling in both the pericalcarine and banks of superior temporal sulcus are shown below.

Pericalcarine				
Pass (no error)	Pass (no error)	Minor overestimation & lingual underestimation	Minor overestimation & cuneus underestimation	Minor overestimation & cuneus underestimation
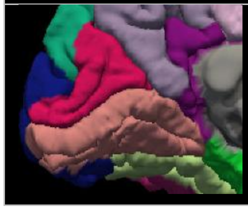	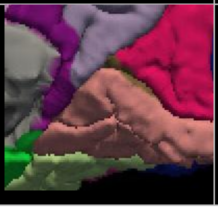	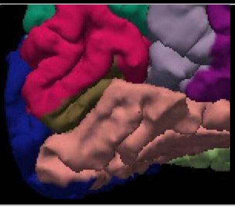	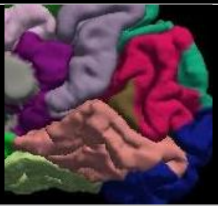	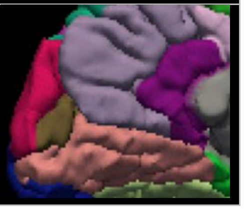

Banks of Superior Temporal Sulcus (FreeSurfer DK ATLAS ONLY not in FastSurfer DKT atlas)		
Minor overestimation of banks and middle temporal.		Banks ok (no error)
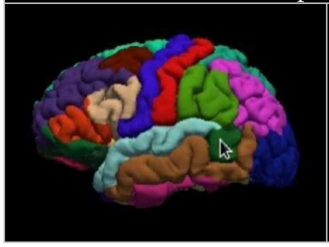	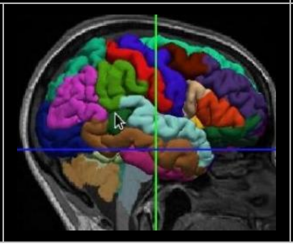	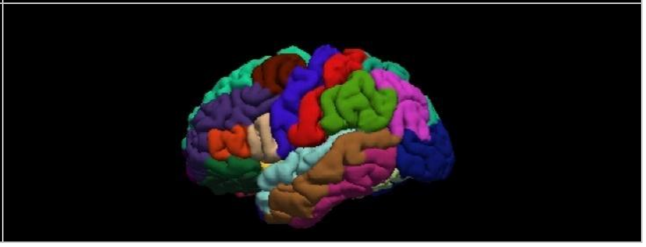

3.3 Classifying Error types

The table below outlines what error ratings should be given to different sized errors. Given that the primary purpose of identifying and classifying cortical parcellation errors is to identify any regions that should be excluded from downstream analyses, we suggest the size of errors that should result in ROI exclusion should be somewhat relative to the size of the ROI itself.

3.3.1 Minor Errors

**MINOR ERRORS**<br>Errors affecting up to 20% of the region (based on visual estimation)
**Minor unconnected** errors - mislabeling, or pial over or underestimation, affecting less than 20% of the region.
Minor unconnected underestimation of the lateral orbitofrontal (dark green), shown across 4 consecutive slices.
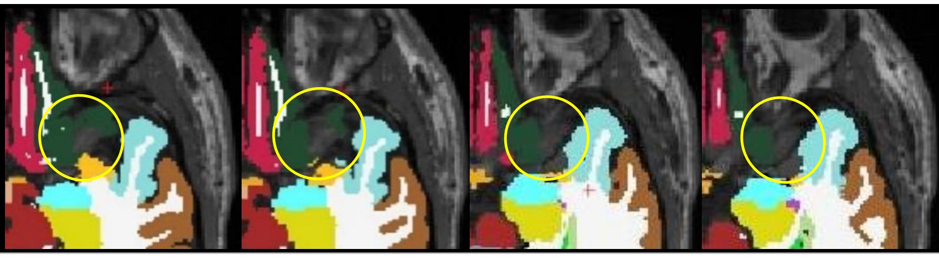
Below: Minor underestimation of the rostral middle frontal (dark purple), shown across 4 sequential slices.
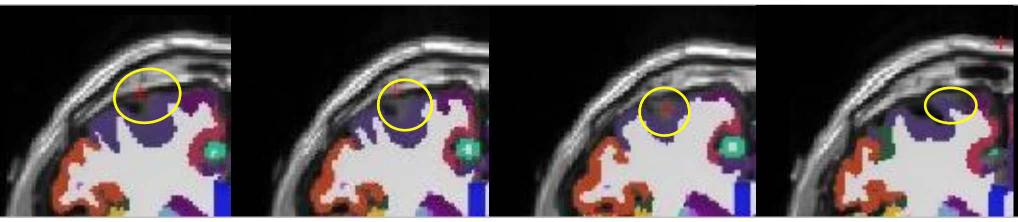
Below: Minor underestimation of the superior temporal (light blue). Error is shown across 4 consecutive slices. The appearance of a slight underestimation of the middle temporal (brown) present in the first 2 slices shown here was deemed an acceptable error and not recorded.

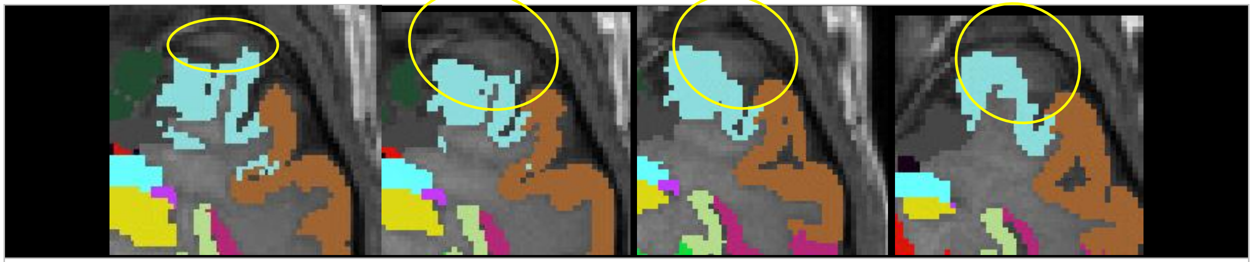

Below: Minor overestimations of the superior/parietal midline are common. In the instance below, the overestimation is restricted to the left hemisphere and so is classed as an unconnected error. In some instances of larger overestimation, the error may encompass both hemispheres and could warrant inclusion as a connected error.

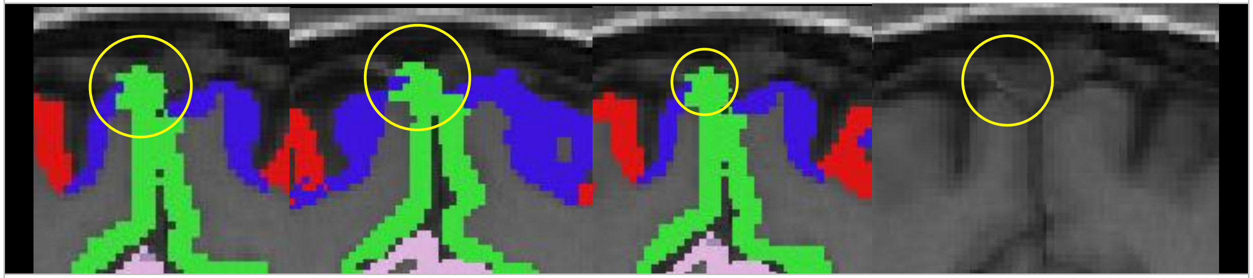

Below is an unconnected minor underestimation of the rostral anterior cingulate (dark purple). Although in the axial view (shown in the two left hand side images) it appears the medial orbital frontal (pink) and superior frontal (green) may be connected to this error, review of the coronal view (shown in the two images on the right hand side) show this error is confined to the rostral anterior cingulate.

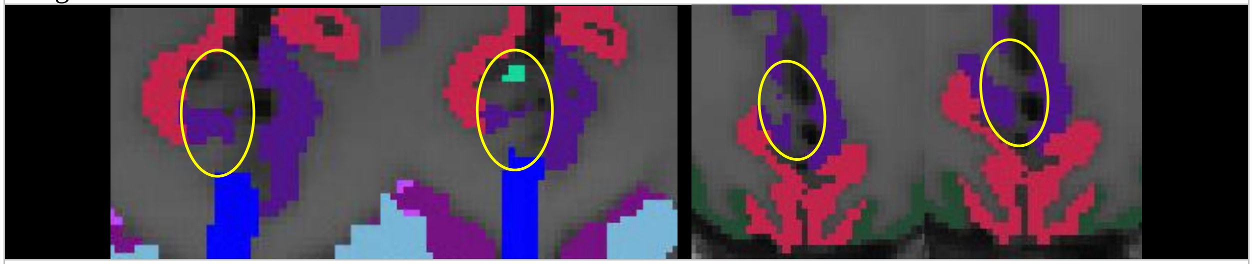

Minor connected errors can remain classified as one minor error if, when added together, they remain affecting less than 20% of the region.

Connected errors at the temporal pole are very common. These errors typically begin with an underestimation of the temporal pole, and since FreeSurfer gives it a grey colour, these errors can be very difficult to discern. It is important to move your cursor within this area and look for when the 'temporal pole' label appears in the information panel in the bottom right to be sure where the parcellation begins and ends. Connected errors in this area can often affect the temporal pole, inferior temporal and middle temporal, as well as the fusiform and superior temporal regions. Due to the frequency of errors occurring within this area, we are more lenient with their classification.

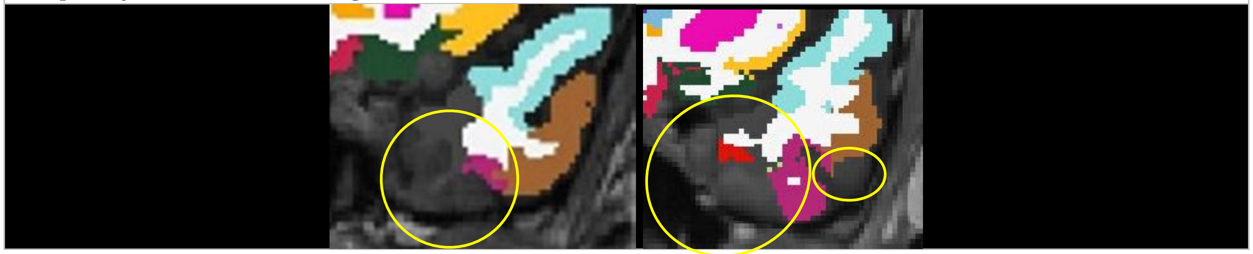

3.3.2 Intermediate Errors

INTERMEDIATE<br>(Errors affecting 20-50% of the region)
If **multiple minor connected errors** persist across slices such that the total affected area may correspond to 20% or more of any one of the affected regions, then it is classified as one intermediate error, though the error is recorded in each region (see section 4).
**Intermediate unconnected errors**
One intermediate unconnected error affecting roughly 20-50% of the Pars Opercularis
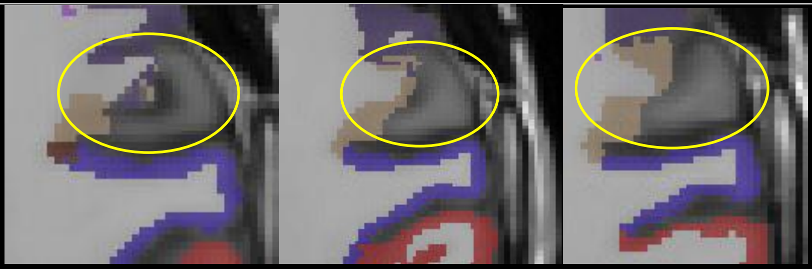
Below: one intermediate unconnected error affecting the postcentral. This example represents a rare case where separate errors can occur in the same ROI.
The postcentral here contains an intermediate underestimation circled in yellow, as well as a minor overestimation circled in blue.
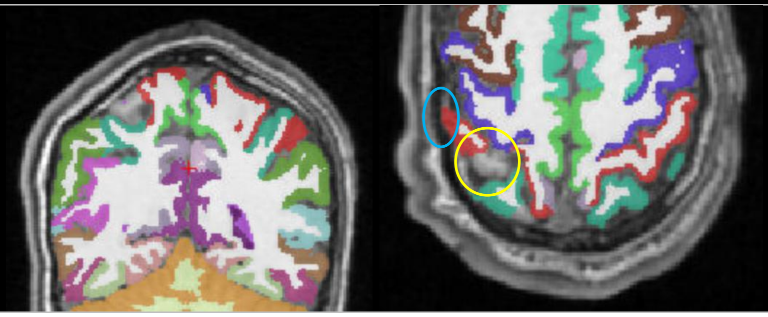

3.3.3 Major Errors

**MAJOR Errors**<br>(Errors affecting greater than 50% of the region)
**Multiple connected errors**<br>Almost every region in the image below is visibly thinner than would be expected. The parcellation does not extend to the outline of the underlying GM, with many of the deeper sulcal folds having not been parcellated, instead the parcellation has clung to the outer surface. When we see multiple major underestimations like this, the image parcellation needs to be failed. It is sufficient to put a major error in only one of the ROIs and then a note or a screenshot to explain the effect across multiple regions.
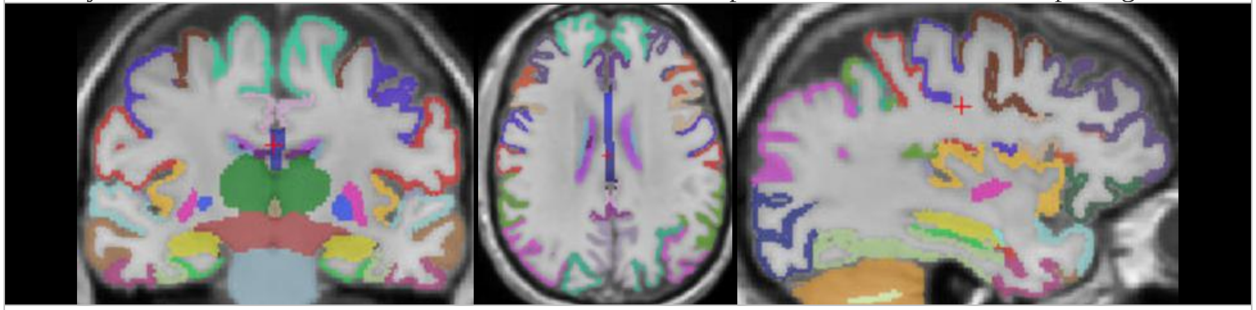
**A large unconnected error affecting greater than 50% of a region**
It is rare, but possible, that a total processing failure such as that below might occur. It would be sufficient to record a major error in only one of the ROIs and then a note or a screenshot explaining the effect across multiple regions. This type of failure may be caused by corruption when running the parcellation and might be avoided/removed by re-running the parcellation.<br>(The image below is taken from the ENQC guide)
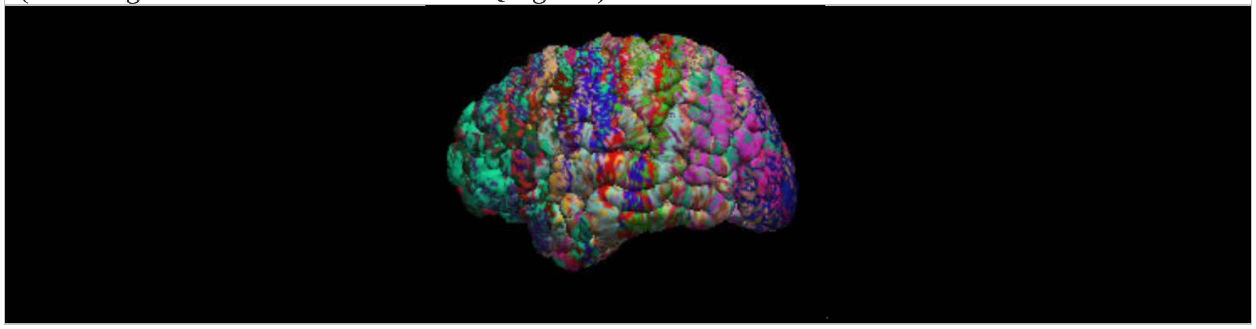

3.3.4 Control Point Errors

**Control Point (CP) errors** (Errors thought to be fixable by adding one or more FreeSurfer control points)		
The images below represent an example of the type of errors that can be fixed according to the FreeSurfer tutorial. These specific CPs only fix WM exclusions or GM mislabeling resulting from missing WM. The tutorial provides other examples of edits that can be made (https://surfer.nmr.mgh.harvard.edu/fswiki/FsTutorial/TroubleshootingData).		
Initial, incorrect parcellation excluding WM in the temporal lobes.	Green dots are the control points added to show Freesurfer where the WM extends to.	Subsequently improved parcellation after the addition of WM control points.
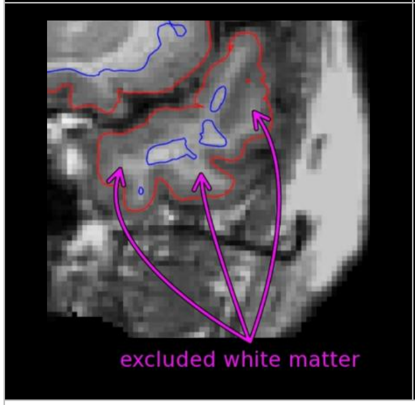	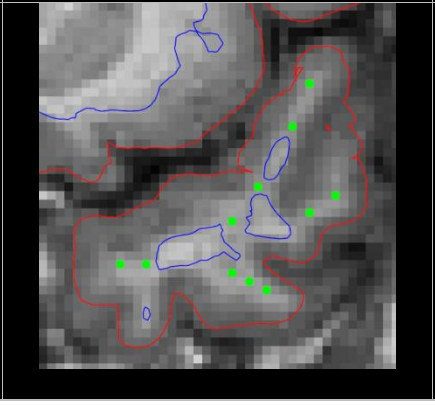	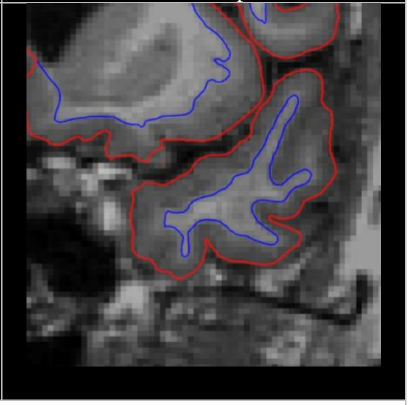
The three images above were sourced from the FreeSurfer Tutorial online (https://surfer.nmr.mgh.harvard.edu/fswiki/FsTutorial/TroubleshootingData).		

<u>4.0 Recording Errors</u>

4.1 EAGLE-I Error Tracker

The overarching aim of conducting detailed QC of parcellation images is to ensure that any identified errors are recorded and can be easily referred to during subsequent analyses. To aid this, we have updated the *ENIGMA Cortical QC Template* which was originally provided alongside the ENQC guide. The main updates include:

1. <u>Separate columns for L/R regions</u>: To aid in data extraction of the frequency of under/overestimations in each specific FreeSurfer Region, the error tracker includes separate columns for each region individually.
2. <u>Separate columns for internal/external view:</u> The addition of separate columns for internal/external provides: a) A constant reminder to review abnormal-appearing regions in both internal & external views, b) An option for recording separate errors identified within the same region, eg: intermediate underestimation of the postcentral alongside a minor overestimation (example image provided on page 14 (intermediate unconnected error)). c) An option for recording the exact same errors observed in different views by merging the internal and external cells for that region, e.g., overestimation of the superior frontal , example image provided on page 17, row 4 of the coding errors table).
3. <u>Automating whole subject QC ratings</u>: columns C-F and K-AB have been locked in the EAGLE-I ET. These columns contain formulas which calculate the number of errors provided across the different brain regions. An overall QC rating for the subject is then assigned to the subject based on the rules and thresholds detailed in the subject QC rating table in section 5.0 of this protocol.

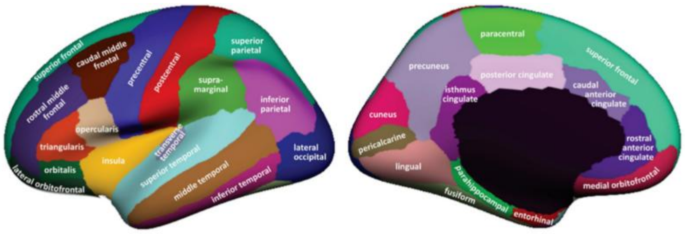

4.2 Code for Recording Errors in Spreadsheet

To simplify data extraction of error type and frequency across regions and images, we have implemented a new code for recording errors. A numerical code denotes type and severity, whilst alphabetical prefixes and suffixes represent connected and control point errors respectively.

QC_Code	Key
	Pass / No Error
-1	Minor Underestimation
1	Minor Overestimation
-2	Intermediate Underestimation
2	Intermediate Overestimation
-3	Major Underestimation
3	Major Overestimation
A	Prefix linking first set of connected errors. Second set would be prefixed B, third set C and so on.
CP	Suffix added to errors thought to be fixable by adding a/multiple control points in freesurfer
	Yellow cell style highlights the Freesurfer common error regions.

The table below provides visual examples of different types of errors, a corresponding description of the error, as well as an example of how that error should be recorded in the EAGLE-I ET.

Image of Error	Error Description	Code to enter Error in Spreadsheet
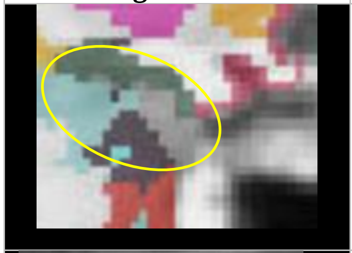	A minor connected error affecting 3 regions: overestimations in the right internal medial orbitofrontal and the right internal lateral orbitofrontal and an underestimation of the right internal superior temporal.	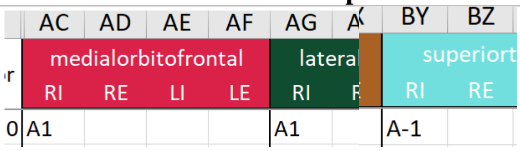
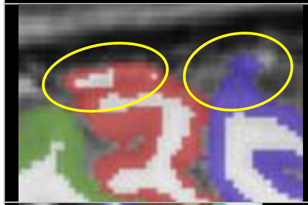	Two minor unconnected errors in both the right internal precentral and the right internal postcentral (these were not obvious in external viewing)	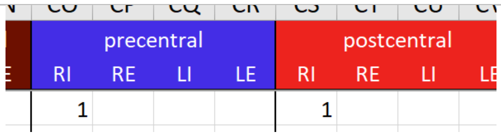
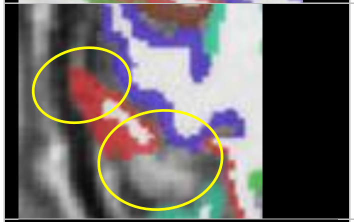	Two unconnected errors in the same ROI. An intermediate underestimation of the postcentral as well as a minor overestimation.	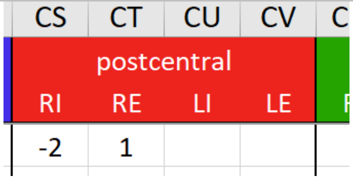
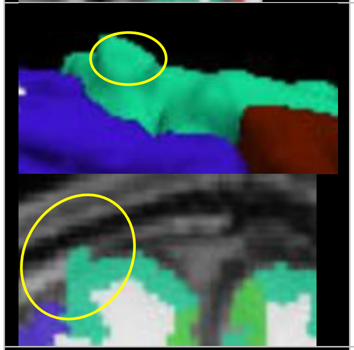	A minor overestimation of the right superior frontal that is clearly visible in both the internal and external views.	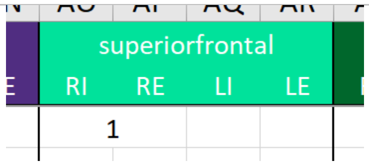
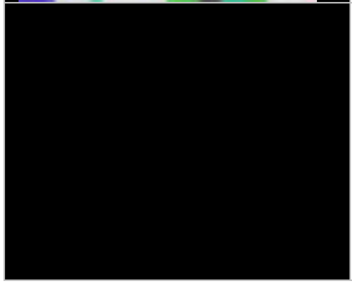	Two separate sets of connected errors. The error "B" involved underestimation of the Right internal precentral and postcentral. The error "C" involved underestimation of the Left internal precentral and postcentral.	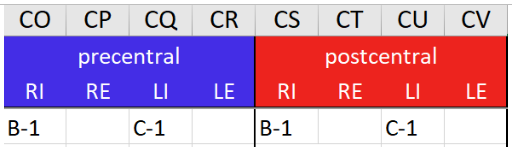

5.0 Image QC Ratings

The table below provides a clear method for counting the number of errors recorded for each participant during QC to systematically determine that participant's overall QC rating and outcome. Within FCER, minor errors are almost universal and are recorded, but do not affect error counts. For example, if a parcellation's only errors were in the FCER then the number of minor errors would be 0, but the FCER errors would be noted in the spreadsheet for future reference. Unless specifically noted as FCER, all other errors refer to those occurring in regions outside of the FCER.

Brain level quality ratings	Error Count Guide	Outcome
Pass	Unlimited minor errors in the FCER<br>AND /OR<br>≤3 minor errors	**Include participant**<br>Exclude FCER if errors are intermediate or major, do not exclude ROIs with minor errors
Minor Error	≤4 minor errors<br>OR<br>1 intermediate error	**Include participant**<br>Include ROIs with minor errors<br>Exclude the ROI with the intermediate error.
Major Error	5 – 7 minor errors<br>OR<br>2 intermediate errors	**Discuss with additional reviewer**<br>If participant is not easily reclassified as minor error or fail then include participant. but, exclude ROIs with intermediate errors
Fail	>7 minor errors<br>OR<br>> 2 intermediate errors<br>OR<br>1 major error	**Exclude whole participant**
Discuss	If any error can potentially be fixed (CP error), the participant should be rated as 'discuss' (see FreeSurfer's troubleshooting guide for error fixing)	After attempting to fix the error/s the participant should have QC redone, and a new rating assigned

Example screenshot of the EAGLE-I ET. Columns G-I count the number of each error type identified in the region columns. Hidden columns C-F combine error counts according to the error count guide above. Brain level quality rating is automatically provided in Column B.

	A	B	G	H	I	J
1<br>2	ImageID	QC Rating	Minor	Intermediate	Major	CPs
3	1	Pass	3	0	0	0
4	2	Minor error	3	1	0	0
5	3	Major error	6	0	0	0
6	4	Major error	7	2	0	0
7	5	Fail	11	0	0	0

5.1 Discussion of CP Fix Errors

1. If the CP Fix Error is a minor error, and its inclusion in the error counts still provides an overall Pass or Minor Error rating for the participant then it may be decided not to go ahead and fix the error. The subject should be given the original rating ensuring the CP Fix error is clearly noted.
2. If the CP Fix Error is minor, and its inclusion results in a higher subject classification (i.e., a subject has 5 minor errors and 1 minor CP error [6 total minor errors -> major error]) then the CP should be fixed and parcellation re-run. A complete new visual QC will need to be conducted.
3. If the CP Fix is an intermediate or major error, the CP should be fixed, parcellation re-run, and a new visual quality check performed.

The rules above have been created with whole brain morphometric analysis in mind. In studies focusing on specific regions of the brains, these rules can be altered such that even minor CP fix errors occurring within brain regions of focus are fixed and parcellation re-run.

6.0 Loading FastSurfer Volumes, Surfaces, and Lesion masks in Freeview

6.1 Loading from Bash Command

##Command to load FastSurfer output, NO LESION MASK##

```
export FREESURFER_HOME=/path to your FreeSurfer
source $FREESURFER_HOME/SetUpFreeSurfer.sh
cd /path_to_your_subject_FS_output_folders
export SUBJECTS_DIR=`pwd`
for SUBJECT in HC_01 HC_02 HC_03; do
vglrun freeview -v ${SUBJECT}/mri/orig.mgz \
$SUBJECT/mri/aparc.DKTatlas+aseg.mapped.mgz:colormap=lut:opacity=0.4 \
-f ${SUBJECT}/surf/lh.pial:annot=aparc.DKTatlas.mapped.annot:visible=0 \
${SUBJECT}/surf/rh.pial:annot=aparc.DKTatlas.mapped.annot:visible=0 \
--viewport 3d
done
```

##Command to load FastSurfer output WITH LESION MASK named “sLesion_mask.mgz” saved in the FastSurfer output mri folder##

```
export FREESURFER_HOME=/path_to_your_FreeSurfer
source $FREESURFER_HOME/SetUpFreeSurfer.sh
cd /path_to_your_subject_FS_output_folders
export SUBJECTS_DIR=`pwd`
for SUBJECT in QA01 QA02 QA03; do
vglrun freeview -v ${SUBJECT}/mri/orig.mgz \
$SUBJECT/mri/aparc.DKTatlas+aseg.mapped.mgz:colormap=lut:opacity=0.4 \
-v $SUBJECT/mri/sLesion_mask.mgz:colormap=binary:opacity=1 \
-f ${SUBJECT}/surf/lh.pial:annot=aparc.DKTatlas.mapped.annot:visible=0 \
${SUBJECT}/surf/rh.pial:annot=aparc.DKTatlas.mapped.annot:visible=0 \
--viewport 3d
done
```

6.2 Manual loading

1. File -> Load Volume -> go to the mri folder within your subjects FastSurfer output, select *orig.mgz* -> Open

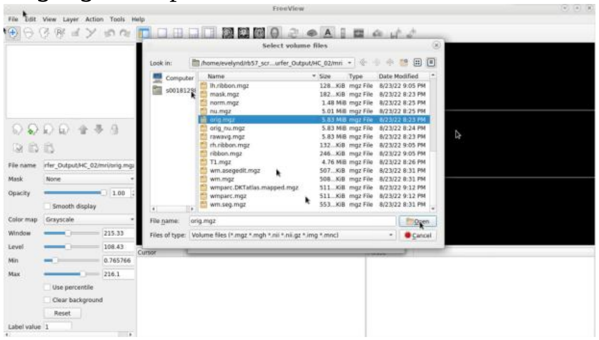

2. File -> Load Volume -> select *aparc.DKTatlas+aseg.mapped.mgz* -> change colourmap to Lookup Table (see screenshot below) -> Open

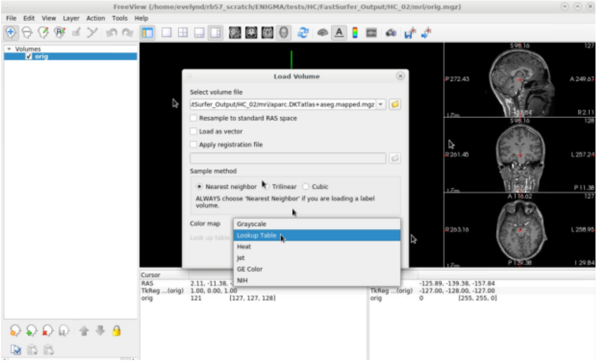

3. File -> Load Surface -> select *lh.pial* -> Open
4. Annotation -> load from file -> select *lh.aparc.DKTatlas.mapped.annot* -> Open

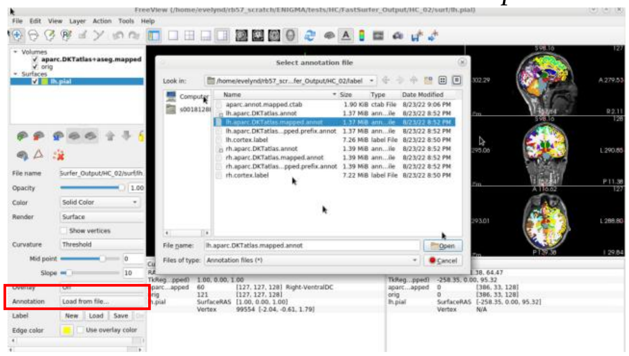

5. File -> Load Surface -> select *rh.pial* -> Open
6. Annotation -> load from file -> select *rh.aparc.DKTatlas.mapped.annot* -> Open

6.3 Turn off WM parcellations (Optional)

Some people find it visually easier to conduct QA with the WM parcellations turned off (this is only an option when running freeview with fastsurfer/7.2.0, as opposed to 6.0). If you would like to turn the WM parcellations off:

Click on your *aparc.DKTatlas+aseg.mapped* volume in the top left -> scroll down to where you see a list of labels -> uncheck labels *2 Left–Cerebral-White-Matter* and *41 Right-Cerebral-White-Matter*

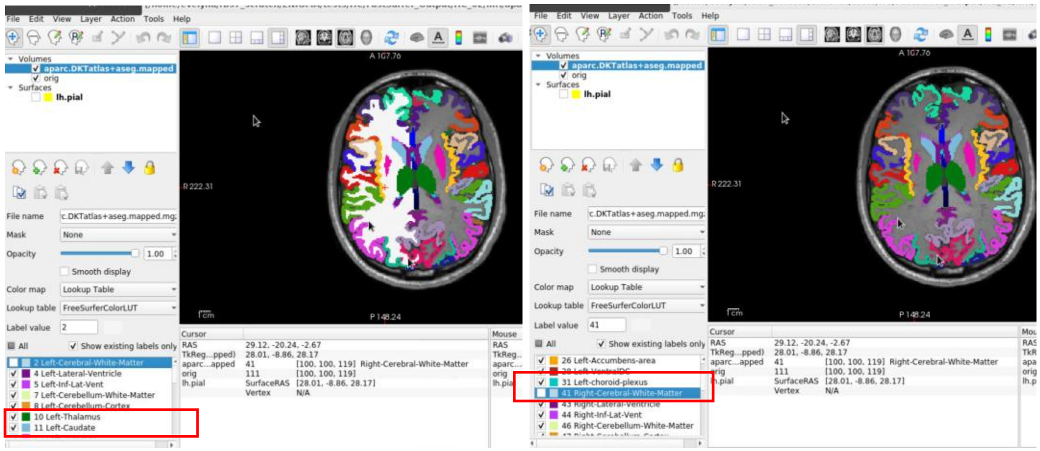

6.4 Loading a Lesion mask on top of FastSurfer parcellations

For clinical populations where large lesions or other pathology has been filled prior to running FastSurfer, we do not check the QA of the filled region as the underlying parcellations should not contribute to any downstream analyses. To ensure we are only conducting QA on the required regions, overlay the lesion mask on top of the cortical parcellation as follows:

1. Ensure *orig.mgz* and *aparc.DKTatlas.aseg*.mapped are already loaded. Click on -> File -> Load new volume -> select *your_lesion_mask* -> Open
2. Opacity -> 1.0
3. Clear Background

(*Please note: the lesion mask in the image below is not actually from the underlying brain. It has been provided here only as an example of how to load a mask. This example will be updated in a later version of this document)

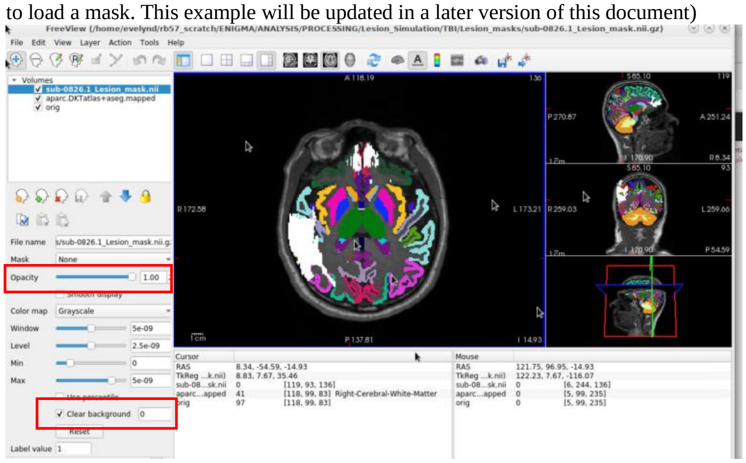

References

Iscan, Z., Jin, T. B., Kendrick, A., Szeglin, B., Lu, H., Trivedi, M., Fava, M., McGrath, P. J., Weissman, M., Kurian, B. T., Adams, P., Weyandt, S., Toups, M., Carmody, T., McInnis, M., Cusin, C., Cooper, C., Oquendo, M. A., Parsey, R. V., & DeLorenzo, C. (2015). Test–retest reliability of freesurfer measurements within and between sites: Effects of visual approval process. *Human Brain Mapping*, *36*(9), 3472–3485. https://doi.org/10.1002/hbm.22856

Klapwijk, E. T., van de Kamp, F., van der Meulen, M., Peters, S., & Wierenga, L. M. (2019). Qoala-T: A supervised-learning tool for quality control of FreeSurfer segmented MRI data. *NeuroImage*, *189*, 116–129. https://doi.org/10.1016/j.neuroimage.2019.01.014

Radwan, A. M., Emsell, L., Blommaert, J., Zhylka, A., Kovacs, S., Theys, T., Sollmann, N., Dupont, P., & Sunaert, S. (2021). Virtual brain grafting: Enabling whole brain parcellation in the presence of large lesions. *NeuroImage*, *229*, 117731. https://doi.org/10.1016/j.neuroimage.2021.117731

Reddy Raamana, P. (2023). VisualQC: Software development kit for medical and neuroimaging quality control and assurance. *Aperture Neuro*, 79. https://doi.org/10.52294/e130fcd2-ce83-4222-856d-c82022013a50
